# The immune landscape of sepsis and using immune clusters for identifying sepsis endotypes

**DOI:** 10.3389/fimmu.2024.1287415

**Published:** 2024-04-19

**Authors:** Guoxing Tang, Ying Luo, Huijuan Song, Wei Liu, Yi Huang, Xiaochen Wang, Siyu Zou, Ziyong Sun, Hongyan Hou, Feng Wang

**Affiliations:** Department of Laboratory Medicine, Tongji Hospital, Tongji Medical College, Huazhong University of Science and Technology, Wuhan, China

**Keywords:** sepsis, immune indicators, endotypes, MDSCs, prediction model

## Abstract

**Background:**

The dysregulated immune response to sepsis still remains unclear. Stratification of sepsis patients into endotypes based on immune indicators is important for the future development of personalized therapies. We aimed to evaluate the immune landscape of sepsis and the use of immune clusters for identifying sepsis endotypes.

**Methods:**

The indicators involved in innate, cellular, and humoral immune cells, inhibitory immune cells, and cytokines were simultaneously assessed in 90 sepsis patients and 40 healthy controls. Unsupervised k-means cluster analysis of immune indicator data were used to identify patient clusters, and a random forest approach was used to build a prediction model for classifying sepsis endotypes.

**Results:**

We depicted that the impairment of innate and adaptive immunity accompanying increased inflammation was the most prominent feature in patients with sepsis. However, using immune indicators for distinguishing sepsis from bacteremia was difficult, most likely due to the considerable heterogeneity in sepsis patients. Cluster analysis of sepsis patients identified three immune clusters with different survival rates. Cluster 1 (36.7%) could be distinguished from the other clusters as being an “effector-type” cluster, whereas cluster 2 (34.4%) was a “potential-type” cluster, and cluster 3 (28.9%) was a “dysregulation-type” cluster, which showed the lowest survival rate. In addition, we established a prediction model based on immune indicator data, which accurately classified sepsis patients into three immune endotypes.

**Conclusion:**

We depicted the immune landscape of patients with sepsis and identified three distinct immune endotypes with different survival rates. Cluster membership could be predicted with a model based on immune data.

## Introduction

Sepsis, one of the leading causes of morbidity and mortality in hospitals, was traditionally considered a systemic inflammatory response syndrome due to infection (1–3). Sepsis is now defined as a life-threatening organ dysfunction caused by a dysregulated host response (4, 5). A recent burden of sepsis report highlights nearly 50 million new cases globally per year (3, 6). Although the prognosis of sepsis varies depending on the different organisms, sites of infection, or underlying host conditions, there are an estimated 10 million deaths each year (3, 6). Despite hundreds of clinical trials conducted, there is currently no single treatment that consistently saves lives in sepsis patients (4, 6).

The dysregulated immune response is described as concurrent hyperinflammation and immune suppression, which is related to many protection mechanisms that become detrimental (4, 6). Among the many mediators implicated in sepsis-associated excessive inflammation, neutrophils, macrophages, cytokines, and coagulation systems are prominently featured (7–10). On the other side, immune suppression, which also involves different cell types, is related to enhanced apoptosis of T cells and increased numbers of inhibitory cells, including regulatory T (Treg) cells and myeloid-derived suppressor cells (MDSCs) (11–14). Generally, longitudinal analyses of immune reactions from early pathogen–host interactions to clinically manifested sepsis in humans are lacking, making the concurrent hyperinflammation and immune suppression during the pathophysiological path of sepsis speculative.

Another core challenge in depicting the immune response of sepsis is the considerable heterogeneity in which the extent of proinflammatory and immunosuppressive responses and their relative contribution to sepsis-associated immunopathology varied between patients (15, 16). Heterogeneity is considered a major factor in the failure of immune modulatory trials in patients with sepsis, and it has been proposed that stratification of patients in subgroups with shared features can improve the effect of therapy, in particular if patient classification is based on characteristics of host response (15–17). Recently, attempts have been made to identify sepsis subgroups with different disease outcomes using clinical, laboratory, and transcriptome data and unbiased computational analysis tools (18–23). In spite of the importance of sepsis subgroup classification in understanding the heterogeneity of patients, stratification of sepsis patients into endotypes based on immune indicators is still rare, and the utility of these subgroups in clinical practice needs to be further determined.

In view of the fact that the immune response is complicated and of key importance in the prognosis of sepsis, we systematically investigated the immune indicators involved in innate, cellular, and humoral immune cells, inhibitory immune cells, as well as cytokines and chemokines simultaneously, in the prognosis of patients with sepsis and bacteremia. Furthermore, unsupervised hierarchical clustering was used to identify clusters of patients with sepsis based on similar immune profiles. Notably, we have not only described the immune landscape of patients with sepsis and bacteremia but also identified three clusters of sepsis patients with different survival rates. Additionally, we build a prediction model by using immune data to enable the stratification of patients into three clusters, which might be useful in standard practice as a convenient tool to identify endotypes in the future.

## Materials and methods

### Study subjects

Between February 2021 and February 2022, patients with positive blood cultures for bacteria who were finally diagnosed with bacteremia or sepsis were recruited from Tongji Hospital (the largest tertiary hospital in central China). Blood culture was performed using an automatic blood culture system, and organisms were identified. Antibiotic susceptibility was carried out using standard microbiological methods. We categorized blood cultures that identified coagulase-negative *Staphylococcus* in only one bottle as contaminated, and consequently, the patients with identified coagulase-negative Staphylococcus were excluded from the study. Another group of healthy controls (HCs) without any clinical symptoms of disease matched for gender and age was randomly selected as the control group. Moreover, another cohort of patients with sepsis enrolled at Sino-French New City Hospital (a branch hospital of Tongji Hospital with 1600 beds) was used to validate the accuracy of the built model. This study was approved by the Ethics Committee of Tongji Hospital, Tongji Medical College, Huazhong University of Science and Technology, Wuhan, China (ID: TJ-IRB20211009).

### Data collection and patient classification

At the time of notification of a positive blood culture, the physiological indicators (body temperature, heart rate, breathing rate, and sequential organ failure assessment (SOFA) score) and routine laboratory results were collected from electronic medical records. The demographic and clinical information was also recorded. The enrolled patients mainly received appropriate antibiotics and symptomatic treatment. The clinical outcome was 30-day all-cause mortality from the day of the first positive culture. Patients with positive blood cultures were categorized into bacteremia and sepsis groups. Bacteremia was defined as the isolation of bacteria from at least one blood culture with a compatible clinical syndrome during a hospital stay. Sepsis was defined as patients who meet the criteria of bacteremia together with an acute change in SOFA score ≥ 2, according to the sepsis-3 definitions (5, 24).

### Flow cytometry analysis

Heparinized blood samples were collected from study participants at the time of notification of a positive blood culture. The absolute numbers of T, B, and NK cells were determined by using TruCOUNT tubes and the BD Multitest 6-color TBNK Reagent Kit (BD Biosciences, San Jose, CA, USA) according to the manufacturer’s instructions. Peripheral blood mononuclear cells (PBMCs) were isolated by using Ficoll–Hypaque density gradients. The 10 cell subsets, including CD4^+^ T cells, CD8^+^ T cells, Treg cells, T helper (Th) cells, follicular helper T (Tfh) cells, B cells, NK cells, monocytes, dendritic cells (DCs), and MDSCs (**Supplementary Table S1**), were detected by flow cytometry. All the staining was blocked using an Fc-blocking buffer, and isotype controls with irrelevant specificities were included as negative controls. The pellets were finally analyzed with a FACSCanto flow cytometer (BD Biosciences). The detailed antibody information is presented in **Supplementary Table S2**. Gating strategies for flow cytometric analysis are shown in **Supplementary Figures S1–S8**.

### Cytokine and chemokine analysis

Peripheral blood samples were collected from study participants, and serum was separated by centrifugation and stored at −80°C until use. The serum concentrations of 24 cytokines and chemokines (CCL2, CCL3, CCL4, CD40L, CXCL10, GM-CSF, granzyme B, IFN-α, IFN-γ, IL-1α, Il-1β, IL-1ra, IL-2, IL-4, IL-6, IL-8, IL-10, IL-12p70, IL-13, IL-15, IL-17, IL-33, PD-L1, and TNF-α) (catalog No. LKTM010; R&D Systems, Minneapolis, MN, USA) were measured by microbead array technology using Luminex 200 system (Luminex, Austin, TX, USA).

### Statistical analysis

Continuous variables were expressed as mean ± standard deviation (SD) or median (interquartile range), and comparisons were performed by using the Mann–Whitney *U* test or one-way ANOVA test when appropriate. Categorical variables were compared using the Chi-square test or Fisher’s exact test. Differences among groups based on immune indicators were also determined by t-distributed stochastic neighbor embedding (t-SNE) analysis with R package “Rtsne”. Cluster analysis of the heat map was performed to identify patients with similar immune patterns by using the R package “pheatmap”, and represented as a dendrogram. Unsupervised k-means cluster analysis of the immune indicator data was used to identify sepsis patient clusters, and the optimal number of clusters was determined using the elbow method with R package “factoextra” and “cluster”. Principal component analysis (PCA) was used to determine major variables between different groups. The prediction model was built using a supervised random forest approach by using the R package “randomForest” and “caret”. The importance of each indicator in the classification of patients was estimated by using the mean decrease in accuracy. Kaplan–Meier curves were used for survival analysis and compared by using the log-rank test. Statistical significance was determined as *p* < 0.05. Statistical analyses were performed using SPSS version 19.0 (SPSS, Chicago, IL, USA), GraphPad Prism 8.0 (San Diego, CA, USA), and R 4.0.3 (R Foundation, Vienna, Austria).

## Results

### The immune landscape of patients with sepsis

A total of 115 patients with positive blood cultures were enrolled, including 25 with bacteremia and 90 with sepsis (24 died, 66 survived). Another 40 healthy individuals were recruited as a control group. Sepsis patients have a median age of 57 years old (IQR: 49–66), with men accounting for 76.67%. The main clinical and demographic characteristics of the participants are presented in **Table 1**. The results of all immune indicators in enrolled individuals are presented in **Supplementary Table S3**.

**Table 1:** The demographic and clinical characteristics of the participants.

	Healthy controls (*n* = 40)	Bacteremia patients (*n* = 25)	Sepsis patients (*n* = 90)
Age [years, median (25th–75th percentiles)]	54 (47–61)	59 (52–63)	57 (49–66)
Males [*n* (%)]	30 (75.00)	20 (80.00)	69 (76.67)
SOFA	/	1 (0–1)	7 (5–10)
Medical department			
Intensive care unit [*n* (%)]	/	3 (12.00)	39 (43.33)
Department of Infectious Diseases [*n* (%)]	/	9 (36.00)	11 (12.22)
Other departments [*n* (%)]	/	13 (52.00)	40 (44.45)
Species			
Gram-positive bacteria [*n* (%)]	/	8 (32.00)	38 (46.67)
Gram-negative bacteria [*n* (%)]	/	17 (64.00)	48 (53.33)
Underlying condition or illness			
Diabetes mellitus [*n* (%)]	/	4 (16.00)	17 (18.89)
Hypertension [*n* (%)]	/	3 (15.00)	15 (16.67)
Solid tumor [*n* (%)]	/	5 (20.00)	7 (7.78)

Data are presented as number (%) or median (25th–75th percentiles). SOFA, sequential organ failure assessment.

**Figure 1:**
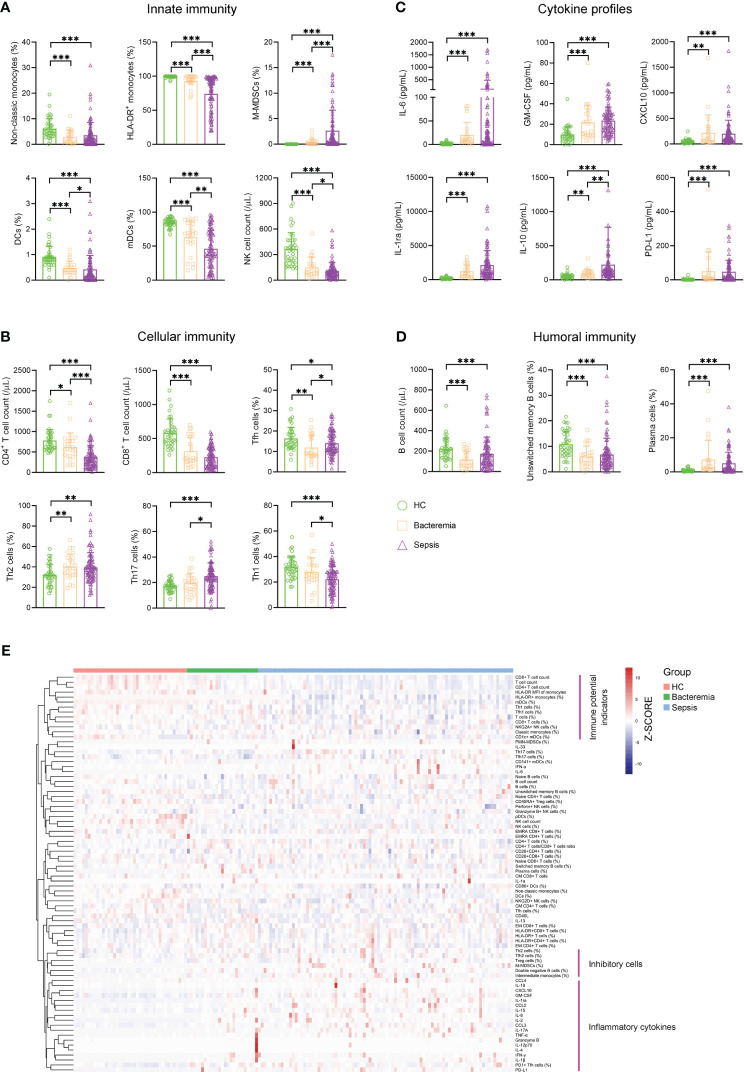
The immune landscape of patients with sepsis. Bar graphs showing the results of representative immune indicators involved in **(A)** innate immunity, **(B)** cellular immunity, **(C)** cytokine profiles, and **(D)** humoral immunity in HC and patients with sepsis and bacteremia. Data are presented as mean and SD. ^*^ *p* < 0.05; ^**^ *p* < 0.01; ^***^ *p* < 0.001. **(E)** Hierarchical cluster analysis of immune indicators in HC (*n* = 40) and patients with sepsis (*n* = 90) and bacteremia (*n* = 25). The pink lines represent typical immune characteristics among different groups. HC, health control.

For innate immunity, the percentage of nonclassic monocytes trended higher in bacteremia and sepsis patients versus HCs. Conversely, bacteremia patients displayed lower HLA-DR expression of monocytes than HCs, and this trend was more pronounced in sepsis patients. Accordingly, the frequency of monocytic MDSCs (M-MDSCs) showed a progressive increase from HCs to bacteremia and sepsis patients. The percentage of DCs, especially the subset of myeloid DCs (mDCs), showed a progressive decrease from HCs to bacteremia and sepsis patients. Although NK cell numbers also showed a decreased trend from HCs to bacteremia and sepsis patients, the expressions of functional markers (NKG2A, perforin, and granzyme B) were comparable among them (**Figure 1A**). For cellular immunity, the numbers of both CD4^+^ and CD8^+^ T cells showed a progressive decrease from HCs to bacteremia and sepsis patients, whereas the frequency of activated HLA-DR^+^CD8^+^ T cells tended to be higher in bacteremia and sepsis patients versus HCs. In addition, sepsis patients demonstrated higher proportions of Th2 and Th17 cells but lower proportions of Th1 and Tfh cells, compared to HCs (**Figure 1B**). For humoral immunity, bacteremia and sepsis patients had a lower number of B cells compared to HCs. In particular, the frequency of unswitched memory B cells was decreased while that of plasma cells was conversely increased in bacteremia and sepsis patients compared to HCs (**Figure 1D**). For cytokine profiles, the levels of both proinflammatory (such as IL-6, GM-CSF, and CXCL10) and anti-inflammatory (such as IL-1ra, IL-10, and PD-L1) cytokines were increased in bacteremia and sepsis patients compared to HCs (**Figure 1C**).

Remarkably, cluster analysis of the heat map showed that low levels of immune potential indicators (e.g., CD4^+^ T-cell count, CD8^+^ T-cell count, and HLA-DR expression on monocytes) coexisted with high levels of inhibitory cells (e.g., Treg cells, M-MDSCs, and Th2 cells), and inflammatory cytokines were the most prominent characteristics in sepsis patients when comparing bacteremia patients or HCs (**Figure 1E**). These data support the impairment of innate and adaptive immunity accompanying increased inflammation in patients with sepsis.

### The differentiation of patients with sepsis and bacteremia

**Figure 2:**
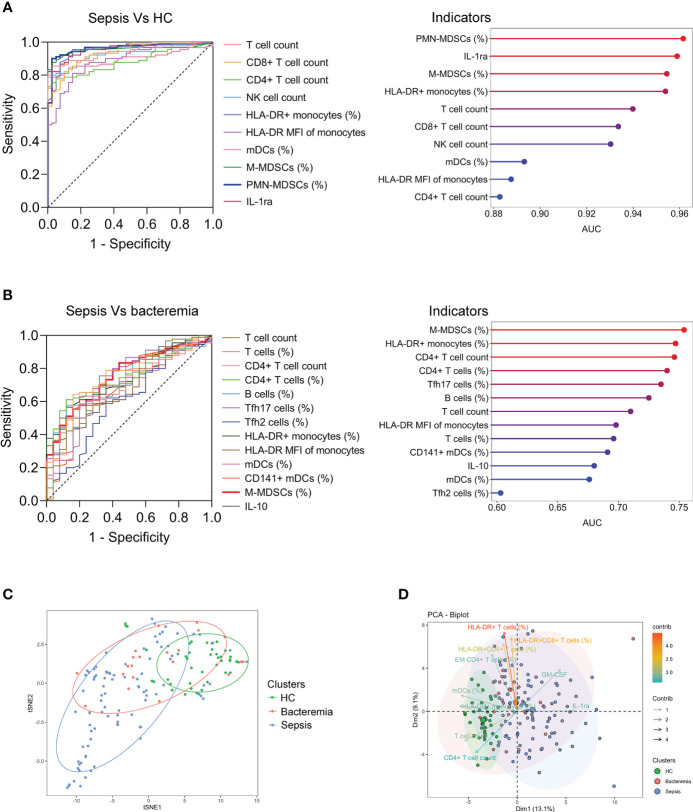
The differentiation of patients with sepsis and bacteremia. **(A)** ROC analysis showing the performance of the top ten indicators in discriminating between sepsis patients and HCs. Cleveland dot plots showing the AUCs of these indicators. **(B)** ROC analysis showing the performance of the indicators (with *p* < 0.01) in discriminating between sepsis and bacteremia patients. Cleveland dot plots showing the AUCs of these indicators. **(C)** The t-SNE analysis using 80 immune indicators to clarify the differences among HC, bacteremia, and sepsis patients. **(D)** The PCA showing the most important variables in the differentiation of patients with sepsis and bacteremia. HC, health control; t-SNE, t-distributed stochastic neighbor embedding; AUC, area under the curve; PCA, principal component analysis.

Many indicators displayed efficient performance in distinguishing between sepsis patients and HCs (**Figure 2A**). Although many indicators like M-MDSCs (%), CD4+ T-cell count, and Tfh17 cells (%) differed significantly between sepsis and bacteremia patients, using a single indicator for distinguishing these two conditions was unsatisfactory. The best indicator was M-MDSCs (%) with an AUC of 0.75 (**Figure 2B**). Expectedly, the t-SNE analysis based on 80 immune indicators showed that sepsis patients could be well distinguished from HCs. However, patients with sepsis and bacteremia showed much overlap and could not be well separated (**Figure 2C**). The subsequent principal component analysis showed that the immune potential indicators (CD4^+^ T-cell count, HLA-DR^+^ monocytes (%), and mDCs (%)) and anti-inflammatory cytokine IL-1ra were the most important variables in HCs and sepsis patients, respectively (**Figure 2D**).

### The immune characteristics of sepsis patients with different outcomes

**Figure 3:**
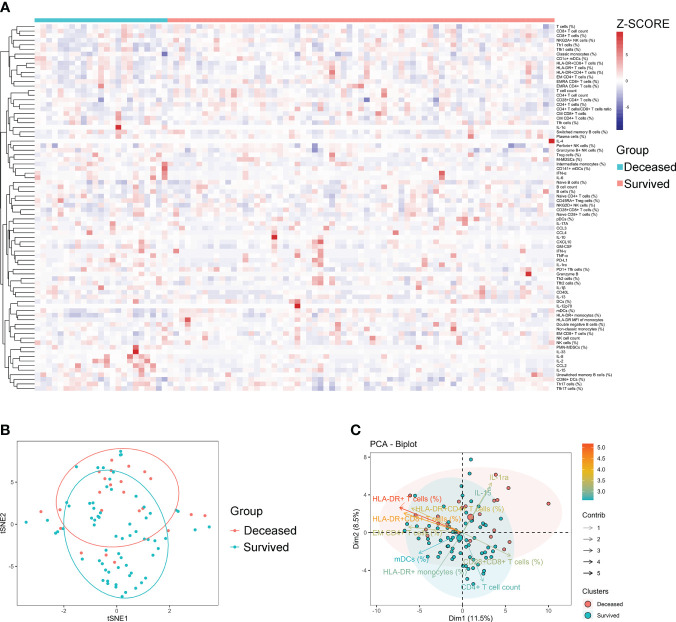
The immune characteristics of sepsis patients with different outcomes. **(A)** Hierarchical cluster analysis of immune indicators in deceased and survived sepsis patients. **(B)** The t-SNE analysis using 80 immune indicators to clarify the difference between deceased (*n* = 24) and survived (*n* = 66) sepsis patients. **(C)** The PCA showing the most important variables in discriminating between deceased and survived patients with sepsis. t-SNE, t-distributed stochastic neighbor embedding; PCA, principal component analysis.

Generally, a few indicators differed significantly between survived and deceased patients with sepsis. Specifically, survived patients displayed higher levels of indicators that represent normal immune potential, such as CD4^+^ T-cell count, HLA-DR^+^ monocytes, and mDCs (%) compared to deceased patients. Conversely, deceased patients demonstrated higher levels of inhibitory cells (M-MDSCs and PMN-MDSCs) and anti-inflammatory cytokines (PD-L1 and IL-1ra) compared to survived patients (**Supplementary Table S4**). Cluster analysis of the heat map did not reveal any pattern of immune characteristics between deceased and survived patients with sepsis (**Figure 3A**). Accordingly, the t-SNE analysis did not advocate the possibility of a combination of these immune indicators for distinguishing deceased from survived patients (**Figure 3B**). Nevertheless, the principal component analysis still showed that the immune potential indicators [CD4^+^ T-cell count, HLA-DR^+^ monocytes (%), and mDCs (%)] and anti-inflammatory cytokine IL-1ra were the most important variables in survived and deceased sepsis patients, respectively (**Figure 3C**).

### Using immune indicators for classifying sepsis endotypes

**Figure 4:**
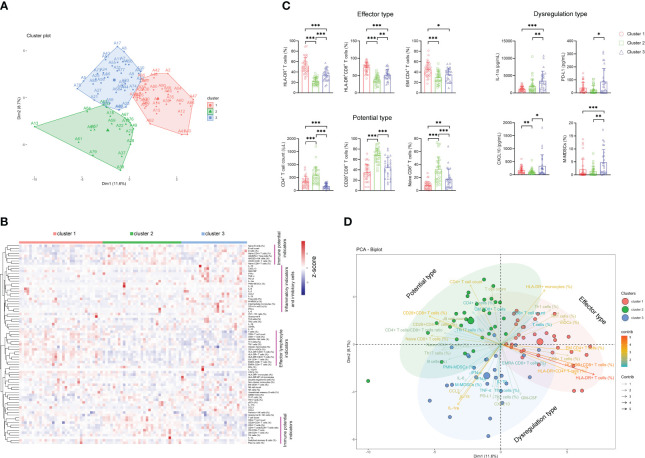
The classification of sepsis endotypes based on immune indicators. **(A)** Unsupervised k-means cluster analysis of 80 immune indicators delivering three distinct clusters of patients with sepsis. **(B)** Hierarchical cluster analysis of immune indicators in sepsis patients grouped by immune cluster (cluster 1, *n* = 33; cluster 2, *n* = 31; cluster 3, *n* = 26). The pink lines represent typical immune characteristics among different clusters of sepsis patients. **(C)** Bar graphs showing the results of representative immune indicators in three clusters of sepsis patients. Data are presented as mean and SD. ^*^ *p* < 0.05; ^**^ *p* < 0.01; ^***^ *p* < 0.001. **(D)** The PCA showing the most important variables in discriminating among three clusters of sepsis patients. PCA, principal component analysis.

Considering the difficulty of using immune indicators for either distinguishing sepsis from bacteremia or predicting the outcome of sepsis, which may be attributed to considerable heterogeneity among sepsis patients, we further determined whether sepsis patients could be classified into different clusters based on these indicators. Notably, unsupervised k-means cluster analysis of 80 immune indicators delivered three distinct clusters of patients with sepsis (**Figure 4A**). Cluster 1 represented 36.7% of the patients with sepsis. Cluster 1 was characterized by an effector phenotype expressed on T cells distinctive from that of the other clusters by virtue of having a high level of HLA-DR on CD4^+^ and CD8^+^ T cells, accompanying increased EM CD4^+^ T-cell frequency (**Figures 4B, C**).

Cluster 2 represented 34.4% of the patients with sepsis. The patients in this cluster had significantly higher levels of immune potential indicators such as CD4^+^ T-cell number, naïve CD8^+^ T-cell percentage, and CD28^+^CD8^+^ T-cell percentage than did the patients in clusters 1 and 3 (**Figures 4B, C**). Cluster 3 represented 28.9% of the patients with sepsis. Cluster 3 was characterized by a dysregulated immune state distinctive from that of the other clusters by virtue of having the highest levels of proinflammatory cytokines (such as CXCL-10 and GM-CSF), anti-inflammatory cytokine (such as IL-1ra and PD-L1), and inhibitory cells (such as M-MDSCs and Treg cells) simultaneously (**Figures 4B, C**). Accordingly, we named clusters 1, 2, and 3 as “effector type”, “potential type”, and “dysregulation type”, respectively, in terms of the most important variables by principal component analysis (**Figure 4D**).

**Figure 5:**
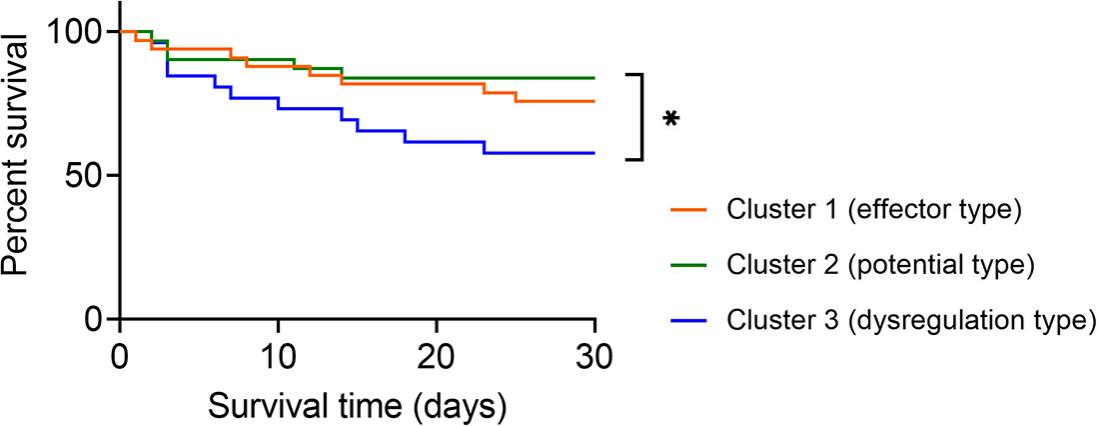
Survival analysis of sepsis patients grouped by immune endotypes. Kaplan–Meier survival curves showing the 30-day survival rates for three clusters of sepsis patients. ^*^ *p* < 0.05 (log-rank test).

Expectedly, the survival rate of sepsis patients in cluster 2 (potential type) reached 83.87% and was the highest among the three clusters. The survival rates in cluster 1 (effector type) and cluster 3 (dysregulation type) were 75.76% and 57.69%, respectively. Notably, sepsis patients in cluster 2 demonstrated significantly higher survival rates than those in cluster 3 (**Figure 5**).

### Prediction model for classifying sepsis endotypes

**Figure 6:**
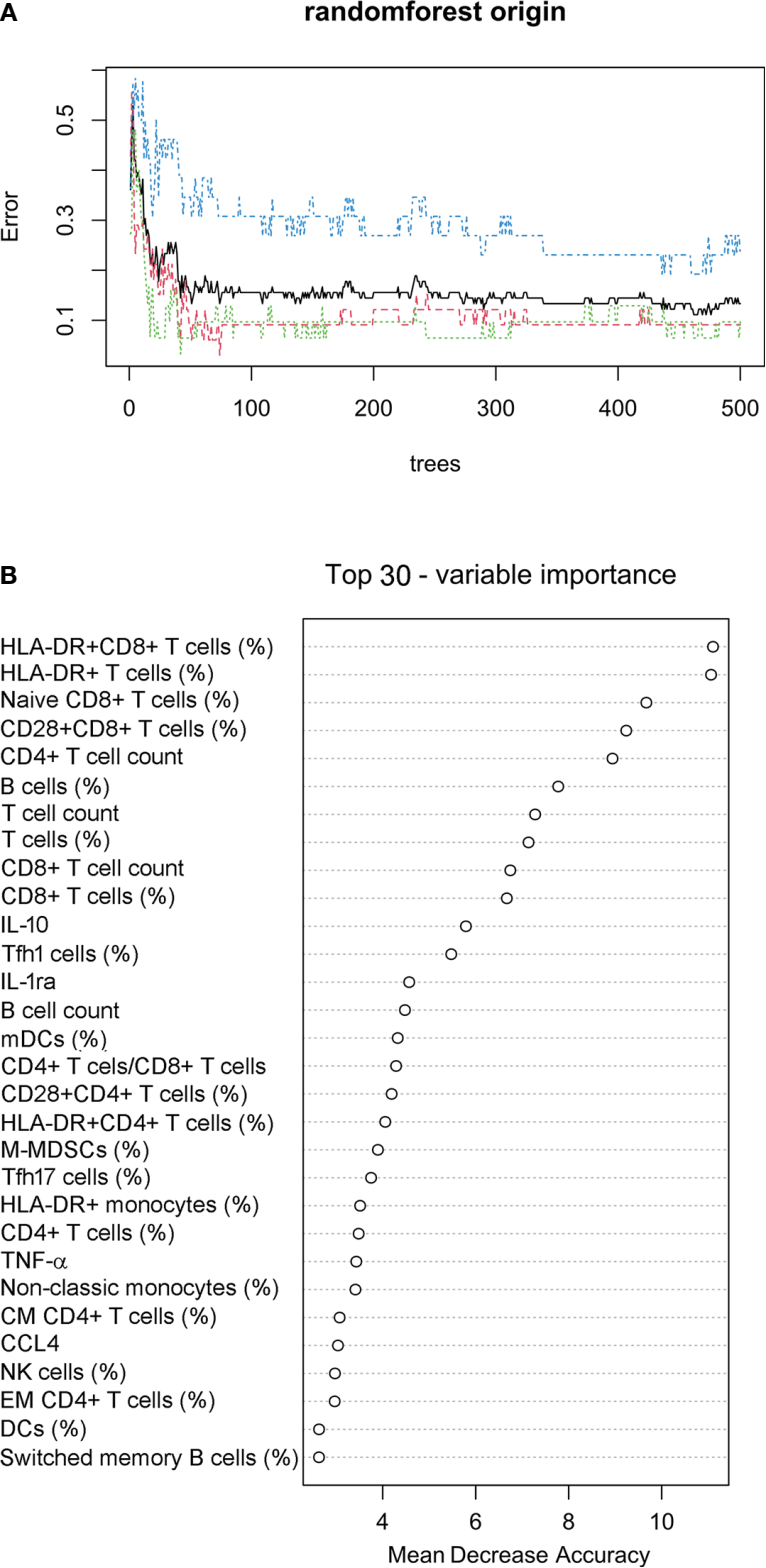
Prediction model for classifying sepsis endotypes. **(A)** The random forest approach was used to build a prediction model for classifying sepsis endotypes (cluster 1, 2, or 3) based on 80 immune indicator data from 90 patients with sepsis. The out-of-bag error estimate for the model was 13.33%. **(B)** Cleveland dot plots showing the top 30 immune indicators sorted by importance for prediction based on the mean decrease in accuracy.

Considering the different survival rates in three sepsis endotypes, we further used the random forest approach to build a prediction model for classifying sepsis endotypes (cluster 1, 2, or 3) based on 80 immune indicator data from 90 patients with sepsis. It was found that after 50 trees, the out-of-bag (OOB) error rate tended to be stable (**Figure 6A**). The top 30 immune indicators were sorted by importance for prediction based on the mean decrease in accuracy (**Figure 6B**). The top 15 indicators were HLA-DR^+^CD8^+^ T cells (%), HLA-DR^+^ T cells (%), Naïve CD8^+^ T cells (%), CD28^+^CD8^+^ T cells (%), CD4^+^ T-cell count, B cells (%), T-cell count, T cells (%), CD8^+^ T-cell count, CD8^+^ T cells (%), IL-10, Tfh1 cells (%), IL-1ra, B cell count, and mDCs (%).

The confusion matrix shows the accuracy of the model built on the 80 immune indicator data measured in 90 patients with sepsis (**Table 2**). Except for cluster 3, the other two clusters had a class error lower than 0.10. Hereafter, the OOB error estimate for the model was 13.33%. Moreover, the built model on the original data set from 90 patients with sepsis was used to predict cluster membership from another cohort of 37 patients with sepsis. The accuracy of the prediction model was 86.5% (95% CI: 71.2%, 95.5%), as shown in **Table 3**. We also attempted to build the model with the top 30 important immune indicators based on the mean decrease in accuracy, and the performance of the model is shown in **Supplementary Table S5**. We evaluated the less complex model using our validation cohort, which demonstrated decreased accuracy (**Supplementary Table S6**).

**Table 2:** The accuracy of the predictive model based on 80 immune indicators measured in 90 patients with sepsis.

Predicted cluster	Original cluster			Class error
	1	2	3	
**1**	30	2	1	0.09
**2**	2	28	1	0.09
**3**	4	2	20	0.23

The out-of-bag error estimate for the model was 13.33%.

**Table 3:** The accuracy of the predictive model based on data from another cohort of 37 patients with sepsis.

Predicted cluster	Original cluster		
	1	2	3
**1**	12	0	0
**2**	1	12	1
**3**	2	1	8

The accuracy of the prediction model was 86.5% (95% CI: 71.2%, = 95.5%).

## Discussion

Currently, sepsis has been defined as life-threatening organ dysfunction caused by the dysregulated or dysfunctional host immune response to infection (25–27). However, the nature and mechanism of immune dysregulation in sepsis still remain ambiguous. In this study, three distinct clusters representing different immune status were identified in sepsis patients, which displayed significantly different survival rates. “Effector type” and “potential type” both signify a normally functioning immune state. In effector-type sepsis patients, T cells exhibit heightened activation, and a larger proportion actively perform their functions, indicating the patient’s body is actively and effectively combating the infection. In potential-type sepsis patients, the percentage of T cells is higher, with an increased presence of natural T cells, suggesting the patient’s body is actively generating immune cells from the bone marrow to combat the infection. Conversely, the “dysregulation type” is characterized by the simultaneous excessive release of inflammatory and anti-inflammatory factors, coupled with a substantial presence of immunosuppressive cells. This complete immune dysfunction results in a very poor prognosis for the patient. The three immune endotypes we defined all exhibited manifestations of immune dysregulation, with the “dysregulation type” being the most severe and correlated with the highest mortality rate. However, despite experts proposing a new definition of sepsis, a clear definition and explanation of “dysregulation” were not provided (6). The immune characteristics associated with the dysregulation type, as proposed in this study, may serve as a manifestation and explanation of immune response dysregulation within the latest definition. The results of this study may, to some extent, reveal the cause of the heterogeneity of sepsis, and the model we have established may aid clinicians in identifying the potential endotype of sepsis before its onset in patients, which allows for precisely selecting immune modulators for the treatment of the disease.

The concept of endotypes has been proposed relatively early in sepsis, with genomics widely applied in defining these endotypes. Brendon et al., utilizing the whole-genome expression profiles of peripheral blood from ICU sepsis patients, employed a combination of unsupervised clustering and machine-learning techniques to categorize sepsis patients into four endotypes. Each endotype corresponds to varying degrees of mortality risk, with new biomarkers defined for each endotype (23). Similarly, Arjun et al., including sepsis patients from both emergency departments and ICUs, conducted transcriptomic sequencing and data mining analysis to classify sepsis patients into five distinct endotypes. They comprehensively characterized the immunological features and mortality risks of these five different endotypes of sepsis patients from an RNA perspective (21). In contrast, our study on sepsis endotypes is based on the protein level. Compared to genomics, protein expression can more accurately reflect the patient’s status, and protein detection is more stable. Our approach serves as a complement to endotype research in sepsis.

The complexity of immune cells involved, concurrent hyperinflammation and immune suppression, and heterogeneity of patients are three major challenges to understanding the immunopathology of sepsis. Many immunologic risk factors are involved in the development of sepsis, among them an increase in a variety of inflammatory cytokines, fewer lymphocytes, and an increase in inhibitory cells such as MDSCs and Treg cells, which are prominent characteristics with a poor prognosis (28–30). Consistent with this notion, we found that immune indicators including proinflammatory cells (monocytes, NK cells, DCs, Th1, Th17, and Tfh cells, as well as CD4^+^ and CD8^+^ T cells), anti-inflammatory cells (MDSCs, Treg cells, and Th2 cells), and inflammatory cytokines were markedly altered in sepsis. Of note, due to patient heterogeneity, some indicators without significant differences between patient groups may also have the potential to classify the disease. For instance, although the activation marker HLA-DR expression on T cells did not show a significant difference between sepsis and bacteremia patients, it could be an important marker for identifying sepsis cluster 1 in this study. Thus, the indicators with no obvious change in sepsis could be meaningful for the classification of different endotypes of the disease.

Regarding immune suppression in sepsis, despite the increase of inhibitory cells, including Treg and MDSCs, as previously mentioned (14, 30, 31), we observed that some anti-inflammatory cytokines such as IL-1ra, PD-L1, and IL-10 were the key mediators in negative regulation of sepsis. In particular, we observed that IL-1ra was one of the most important variables in clusters of patients with immune suppression, indicating the superior role of IL-1ra in reflecting immune suppression than other anti-inflammatory cytokines. Moreover, given that the most prominent immune characteristic of sepsis is the dysregulated immune response (11, 12, 32), our results are in line with previous reports showing that the concurrent hyperinflammation and immunosuppression (cluster 3) was the most important sepsis endotype with the lowest survival rate.

Several issues deserve mention. First, the interpretation of our findings might be limited by the sample size and specific bacterial organisms. Further validation with a large clinical cohort is necessary; stratified analysis based on the specific pathogen type is necessary; and the results of this study may not extrapolate to patients with virus- or fungus-related sepsis. Second, the longitudinal analysis of patients with sepsis is difficult due to the broad heterogeneity of patients. The endotypes of patients could also be switched. Third, given that blood samples were collected from study participants at the time of notification of a positive blood culture, the previous empirical antibiotic treatment might impact the results of immune indicators. Fourth, we did compare neutrophils among the three groups. However, the performance of neutrophils was found to be inferior to that of PMN-MDSCs. Furthermore, there is a strong correlation between neutrophils and PMN-MDSCs (**Supplementary Figure S9**). We opted for a ready-made reagent kit for cytokine analysis, which led to the omission of several well-recognized cytokines associated with sepsis progression, such as CXCL-1, IL-18, IL-26, and IL-27.

Taken together, we have described the immune landscape of sepsis patients by systemically analyzing immune cells and their mediators. This study has not only classified sepsis patients into three immune endotypes with different outcomes but also established a prediction model enabling the stratification of patients into different endotypes, which is of potential value in selecting immune modulators for sepsis treatment.

## Data availability statement

The original contributions presented in the study are included in the article/**Supplementary Material**. Further inquiries can be directed to the corresponding authors.

## Ethics statement

This study was approved by the Ethics Committee of Tongji Hospital, Tongji Medical College, Huazhong University of Science and Technology, Wuhan, China (ID: TJ-IRB20211009). The studies were conducted in accordance with the local legislation and institutional requirements. The participants provided their written informed consent to participate in this study.

## Author contributions

GT: Writing – original draft, Data curation, Visualization, Investigation. YL: Writing – original draft, Visualization. HS: Writing – original draft, Software. WL: Writing – original draft, Investigation. YH, XW and SZ: Writing – original draft, Data curation. ZS: Writing – original draft, Funding acquisition. HH: Writing – original draft, Methodology. FW: Writing – review & editing.
