# Supplementary material for: The immune landscape of sepsis and using immune clusters for identifying sepsis endotypes
Source: Front Immunol. 2024 Apr 19;15:1287415. doi: 10.3389/fimmu.2024.1287415 (PMC11066285; doi:10.3389/fimmu.2024.1287415)
Supplement: Supplementary file 1 [file DataSheet_1.docx]

| **Supplementary Table 1**. **The detailed ten immune cell subsets** | |
| --- | --- |
| The immune cells | Subsets |
| (1) CD4^+^ T cells | CD28^+^CD4^+^ T cells |
|  | HLA-DR^+^CD4^+^ T cells |
|  | naive CD4^+^ T cells (CD45RA^+^CCR7^+^) |
|  | central memory (CM) CD4^+^ T cells (CD45RA^-^CCR7^+^) |
|  | effector memory (EM) CD4^+^ T cells (CD45RA^-^CCR7^-^) |
|  | terminally differentiated effector memory (EMRA) CD4^+^ T cells (CD45RA^+^CCR7^-^) |
| (2) CD8^+^ T cells | CD28^+^CD8^+^ T cells |
|  | HLA-DR^+^CD8^+^ T cells |
|  | naive CD8^+^ T cells (CD45RA^+^CCR7^+^) |
|  | central memory (CM) CD8^+^ T cells (CD45RA^-^CCR7^+^) |
|  | effector memory (EM) CD8^+^ T cells (CD45RA^-^CCR7^-^) |
|  | terminally differentiated effector memory (EMRA) CD8^+^ T cells (CD45RA^+^CCR7^-^) |
| (3) Treg cells (CD4^+^CD25^high^CD127^low^） | CD45RA^+^ naive Tregs |
| (4) T helper (Th) cells (CD4^+^CD25^low^CD127^high^CD45RA^-^CXCR5^-^) | Th1 cells (CXCR3^+^CCR6^-^ Th) |
|  | Th2 cells (CXCR3^-^CCR6^-^ Th) |
|  | Th17 cells (CXCR3^-^CCR6^+^ Th) |
| (5) Follicular helper T (Tfh) cells (CD4^+^CD25^low^CD127^high^CD45RA^-^CXCR5^+^) | Tfh1 cells (CXCR3^+^CCR6^-^ Tfh) |
|  | Tfh2 cells (CXCR3^-^CCR6^-^ Tfh) |
|  | Tfh17 cells (CXCR3^-^CCR6^+^ Tfh) |
|  | PD1+ Tfh cells |
| (6) CD19^+^ B cells | IgD^+^CD27^-^ naive B cells |
|  | IgD^+^CD27^+^ unswitched memory B cells |
|  | IgD^-^CD27^+^ switched memoryy B cells |
|  | IgD^-^CD27^-^ double negative B cells |
|  | CD27^+^CD38^high^ plasma cells |
| (7) CD3^-^CD56^+^ NK cells | NKG2A^+^ NK cells |
|  | NKG2D^+^ NK cells |
|  | Perforin^+^ NK cells |
|  | Granzyme B^+^ NK cells |
| (8) Monocytes | HLA-DR^+^CD14^++/+^ monocytes |
|  | CD16^-^CD14^++^ classic monocytes |
|  | CD16^+^CD14^++^ intermediate monocytes |
|  | CD16^+^CD14^+^ non-classic monocytes |
| (9) DCs (CD45^+^Lin^-^HLA-DR^+^) | CD86^+^ DCs |
|  | CD123^+^ plasmacytoid DCs (pDCs) |
|  | CD11c^+^ myeloid DCs (mDCs) |
|  | CD1c^+^ mDCs |
|  | CD141^+^ mDCs |
| (10) MDSCs | CD14^+^CD15^-^CD16^-^HLA-DR^-/low^CD11b^+^CD33^+^ monocytic MDSCs (M-MDSCs) |
|  | CD15^+^CD14^-^CD16^-^HLA-DR^-/low^CD11b^+^CD33^+^ polymorphonuclear MDSCs (PMN-MDSCs) |
| Treg, regulatory T; DCs, dendritic cells; MDSCs, myeloid-derived suppressor cells. | |

| **Supplementary Table 2.** **The detailed antibody information used** | | |
| --- | --- | --- |
| Reagent name | Manufacturer | Catalog |
| anti-CD45-PerCP (2D1) | BD | 664934 |
| anti-CD3-APC-H7 (SK7) | BD | 663490 |
| anti-CD4-V450 (RPA-T4) | BD | 560345 |
| anti-CD8-PE-Cy7 (SK1) | BD | 335822 |
| anti -HLA-DR-APC (L243 (G46-6) ) | BD | 665330 |
| anti-CD28-PE (L293) | BD | 662797 |
| anti-CD4-BV510 (SK3) | BD | 562970 |
| anti-CD45RA-FITC (L48) | BD | 662840 |
| anti-CD25-APC (2A3) | BD | 662525 |
| anti-CD127-BV421 (HIL-7R-M21) | BD | 562436 |
| anti-CCR7-PE (150503) | BD | 560765 |
| anti-CD4-APC-Cy7 (SK3) | BD | 341115 |
| anti-CD45RA-BV510 (HI100) | BD | 563031 |
| anti-CXCR5-BB515 (RF8B2) | BD | 564624 |
| anti-CXCR3-PE-Cy7 (1C6/CXCR3) | BD | 560831 |
| anti-CCR6-PerCP-Cy5.5 (11A9) | BD | 560467 |
| anti-PD-1-BV605 (EH12.1) | BD | 563245 |
| anti-CD45-V500-C (2D1) | BD | 662912 |
| anti-CD19-PE-Cy7 (SJ25C1) | BD | 341113 |
| anti-IgD-APC (IA6-2) | BD | 561303 |
| anti-CD27-PerCP-Cy5.5 | BD | 662839 |
| anti-CD38-FITC (HB-7) | BD | 340909 |
| anti-CD24-PE (ML5) | BD | 555428 |
| anti-CD56-PE (NCAM16.2) | BD | 652825 |
| anti-NKG2D-FITC (1D11) | Biolegend | 320820 |
| anti-NKG2A-V450 (131411) | BD | 747924 |
| anti-CD107a-BV605 (H4A3) | Biolegend | 328634 |
| anti-Perforin-PE-Cy7 (B-D48) | Biolegend | 353316 |
| anti-IFN-γ-APC (B27) | BD | 554702 |
| anti-Granzyme B-BV510 (GB 11) | BD | 563388 |
| anti-CD16-PE (B73.1) | BD | 652824 |
| anti-CD14-APC (MφP9) | BD | 652833 |
| anti-HLA-DR-FITC (LN3) | Biolegend | 327006 |
| Human Lineage Cocktail 4（Lin） | BD | 562722 |
| anti -HLA-DR-APC (L243(G46-6)) | BD | 665330 |
| anti-CD86-PerCP (IT2.2) | Biolegend | 305420 |
| anti-CD11c-PE (3.9) | Biolegend | 301606 |
| anti-CD123-BV605 (6H6) | Biolegend | 306026 |
| anti-CD1c-BV421 (L161) | Biolegend | 331526 |
| anti-CD141-PE-Cy7 (M80) | Biolegend | 344110 |
| anti-CD3-FITC (SK7) | BD | 349201 |
| anti-CD19-FITC (HIB19) | BD | 555412 |
| anti-CD56-FITC (HCD56) | Biolegend | 318304 |
| anti-CD16-PE (3G8) | Biolegend | 302008 |
| anti-CD11b-BV605 (ICRF44) | Biolegend | 562721 |
| anti-CD33-PE-Cy7 (WM53) | Biolegend | 303434 |
| anti-CD14-APC-Cy7 (MφP9) | BD | 333951 |
| anti-CD15-V450 (MMA) | BD | 663504 |

| **Supplementary Table 3. The results of immune indicators in enrolled individuals.** | | | | | | |
| --- | --- | --- | --- | --- | --- | --- |
|  | HCs (n = 40) | Bacteremia (n = 25) | Sepsis (n = 90) | p (HCs vs bacteremia) | p (HCs vs sepsis) | p (bacteremia vs sepsis) |
| WBC count (×109/L) | 5.45 (5.04-6.08) | 9.34 (6.11-12.96) | 11.43 (7.47-15.72) | <0.001 | <0.001 | 0.123 |
| NEU percentage (%) | 57.65 (51.75-62.40) | 75.30 (71.50-83.10) | 85.05 (78.15-91.40) | <0.001 | <0.001 | 0.003 |
| NEU count (×109/L) | 3.14 (2.63-3.73) | 7.40 (4.32-10.10) | 9.15 (6.25-14.34) | <0.001 | <0.001 | 0.050 |
| LYM percentage (%) | 33.65 (29.13-38.03) | 12.80 (10.90-18.10) | 7.80 (4.40-12.15) | <0.001 | <0.001 | <0.001 |
| LYM count (×109/L) | 1.92 (1.62-2.12) | 1.21 (0.97-1.46) | 0.88 (0.52-1.20) | <0.001 | <0.001 | 0.001 |
| MON percentage (%) | 6.70 (5.70-8.03) | 7.30 (5.20-8.40) | 5.45 (3.55-9.38) | 0.666 | 0.084 | 0.061 |
| MON count (×109/L) | 0.37 (0.31-0.45) | 0.68 (0.44-0.94) | 0.62 (0.38-0.90) | <0.001 | <0.001 | 0.517 |
| EOS percentage (%) | 1.60 (1.08-2.43) | 0.70 (0.40-1.90) | 0.20 (0.00-0.78) | 0.047 | <0.001 | 0.002 |
| EOS count (×109/L) | 0.10 (0.06-0.15) | 0.08 (0.04-0.20) | 0.02 (0.00-0.06) | 0.295 | <0.001 | 0.003 |
| BOS percentage (%) | 0.30 (0.20-0.50) | 0.20 (0.10-0.30) | 0.20 (0.10-0.20) | 0.012 | <0.001 | 0.097 |
| BOS count (×109/L) | 0.02 (0.01-0.03) | 0.02 (0.01-0.03) | 0.02 (0.01-0.03) | 0.622 | 0.529 | 0.321 |
| RBC (×1012/L) | 4.72 (4.52-5.16) | 3.36 (3.07-3.90) | 3.02 (2.57-3.46) | <0.001 | <0.001 | 0.004 |
| Hb (g/L) | 142.00 (136.75-161.25) | 99.00 (83.00-112.00) | 91.00 (76.25-101.75) | <0.001 | <0.001 | 0.085 |
| PLT (×109/L) | 235.50 (202.75-264.25) | 261.00 (171.00-312.00) | 87.00 (45.25-176.50) | 0.604 | <0.001 | <0.001 |
| ALT (U/L) | 16.50 (11.00-25.00) | 28.00 (17.00-39.00) | 24.00 (12.00-48.00) | 0.007 | 0.019 | 0.553 |
| AST (U/L) | 18.50 (16.00-23.00) | 31.00 (19.00-50.00) | 39.50 (19.00-59.75) | <0.001 | <0.001 | 0.435 |
| TP (g/L) | 74.55 (71.78-75.90) | 61.70 (55.30-67.00) | 57.55 (52.80-62.93) | <0.001 | <0.001 | 0.152 |
| ALB (g/L) | 45.40 (43.43-47.60) | 29.50 (27.40-32.60) | 29.90 (27.25-32.35) | <0.001 | <0.001 | 0.763 |
| GLB (g/L) | 29.00 (26.98-30.80) | 30.70 (28.10-35.50) | 27.95 (24.03-31.55) | 0.042 | 0.262 | 0.017 |
| A/G | 1.55 (1.48-1.71) | 0.97 (0.80-1.10) | 1.06 (0.90-1.32) | <0.001 | <0.001 | 0.117 |
| TBil (mmol/L) | 9.40 (7.10-12.70) | 9.90 (6.40-18.10) | 24.35 (11.58-52.60) | 0.462 | <0.001 | 0.002 |
| DBil (mmol/L) | 3.35 (2.70-4.35) | 5.60 (3.50-13.60) | 15.40 (6.80-45.93) | 0.004 | <0.001 | 0.004 |
| IBil (mmol/L) | 6.00 (4.33-8.23) | 3.90 (2.70-5.90) | 6.80 (4.50-11.08) | 0.016 | 0.049 | <0.001 |
| LDH (U/L) | 178.50 (158.50-196.00) | 176.00 (172.00-250.00) | 274.00 (207.25-394.00) | 0.042 | <0.001 | 0.001 |
| urea (mmol/L) | 5.10 (4.49-5.80) | 6.90 (3.56-10.33) | 12.89 (8.51-21.95) | 0.082 | <0.001 | <0.001 |
| Cre (mmol/L) | 73.00 (61.00-81.00) | 68.00 (46.00-86.00) | 111.50 (60.25-220.00) | 0.562 | <0.001 | 0.001 |
| uric acid (mmol/L) | 301.50 (255.50-352.75) | 180.00 (145.00-313.00) | 240.00 (156.85-398.68) | 0.001 | 0.069 | 0.091 |
| PT (s) | 12.80 (12.40-13.13) | 14.00 (13.50-14.70) | 16.00 (14.55-17.28) | <0.001 | <0.001 | <0.001 |
| PA (%) | 104.00 (97.75-114.50) | 88.00 (79.00-96.00) | 68.50 (59.25-80.50) | <0.001 | <0.001 | <0.001 |
| INR | 0.98 (0.93-1.00) | 1.08 (1.03-1.16) | 1.27 (1.14-1.41) | <0.001 | <0.001 | <0.001 |
| FIB (g/L) | 2.92 (2.57-3.25) | 4.36 (3.39-5.17) | 3.62 (2.51-5.19) | <0.001 | 0.014 | 0.135 |
| APTT (s) | 35.35 (32.78-37.30) | 43.50 (39.60-47.50) | 43.10 (39.30-50.48) | <0.001 | <0.001 | 0.380 |
| TT (s) | 17.00 (16.60-17.60) | 16.90 (16.00-17.40) | 17.15 (15.80-18.65) | 0.495 | 0.630 | 0.429 |
| T cell count (/ml) | 1329.00 (1145.38-1567.00) | 805.08 (624.72-1411.00) | 587.75 (328.66-851.56) | <0.001 | <0.001 | 0.001 |
| T cells (%) | 70.98 (65.75-76.82) | 77.78 (72.07-81.22) | 68.00 (57.63-77.30) | 0.014 | 0.193 | 0.003 |
| CD8+ T cell count (/ml) | 562.42 (449.62-639.83) | 211.11 (170.51-530.00) | 194.00 (93.48-344.62) | <0.001 | <0.001 | 0.068 |
| CD8+ T cells (%) | 27.76 (23.49-32.25) | 24.81 (16.46-29.00) | 22.39 (15.59-28.31) | 0.042 | 0.003 | 0.786 |
| CD4+ T cell count (/ml) | 715.50 (594.25-900.75) | 560.90 (395.99-774.00) | 314.33 (185.53-481.58) | 0.010 | <0.001 | <0.001 |
| CD4+ T cells (%) | 37.56 (33.23-43.40) | 50.48 (43.76-55.02) | 37.91 (28.07-49.02) | <0.001 | 0.978 | <0.001 |
| NK cell count (/ml) | 355.00 (215.50-467.03) | 100.00 (71.42-189.90) | 86.00 (45.80-119.67) | <0.001 | <0.001 | 0.042 |
| NK cells (%) | 16.16 (11.32-24.14) | 10.93 (6.01-15.89) | 9.36 (6.04-15.14) | 0.014 | <0.001 | 0.567 |
| B cell count (/ml) | 206.62 (154.29-278.50) | 96.32 (57.18-191.95) | 133.00 (71.25-230.75) | <0.001 | 0.001 | 0.190 |
| B cells (%) | 10.54 (8.49-12.84) | 8.37 (4.59-14.31) | 17.16 (9.93-24.87) | 0.299 | <0.001 | 0.001 |
| CD4+ T cells/CD8+ T cells | 1.41 (1.11-1.68) | 2.11 (1.35-3.15) | 1.83 (1.01-2.74) | 0.001 | 0.024 | 0.124 |
| HLA-DR+CD4+ T cells (%) | 17.20 (12.88-24.33) | 21.20 (14.30-31.10) | 17.10 (12.73-22.75) | 0.217 | 0.644 | 0.103 |
| CD28+CD4+ T cells (%) | 94.40 (89.15-95.95) | 96.40 (89.80-98.90) | 95.95 (92.13-98.60) | 0.177 | 0.008 | 0.697 |
| HLA-DR+CD8+ T cells (%) | 46.00 (34.68-62.00) | 61.40 (49.60-73.80) | 53.55 (41.68-72.63) | 0.012 | 0.077 | 0.184 |
| CD28+CD8+ T cells (%) | 50.00 (39.85-71.03) | 47.60 (36.80-66.60) | 49.25 (32.38-67.53) | 0.403 | 0.277 | 0.818 |
| HLA-DR+ T cells (%) | 31.45 (24.40-40.85) | 37.40 (29.80-46.20) | 33.55 (23.75-47.15) | 0.147 | 0.542 | 0.376 |
| CM CD8+ T cells (%) | 3.70 (2.33-5.90) | 5.50 (2.40-7.20) | 2.85 (1.53-4.58) | 0.496 | 0.031 | 0.043 |
| naive CD8+ T cells (%) | 13.75 (7.95-26.13) | 13.10 (5.30-20.20) | 12.95 (6.70-26.88) | 0.808 | 0.852 | 0.836 |
| EM CD8+ T cells (%) | 41.00 (32.68-52.30) | 54.50 (41.90-64.00) | 43.45 (33.98-53.45) | 0.033 | 0.648 | 0.033 |
| EMRA CD8+ T cells (%) | 32.85 (22.48-42.95) | 24.50 (14.20-30.10) | 33.75 (19.70-45.30) | 0.029 | 0.772 | 0.028 |
| CM CD4+ T cells (%) | 26.45 (22.05-33.20) | 24.50 (19.40-31.80) | 26.10 (22.00-31.30) | 0.094 | 0.469 | 0.259 |
| naive CD4+ T cells (%) | 28.00 (23.55-38.45) | 38.50 (25.20-47.20) | 32.20 (23.35-46.53) | 0.151 | 0.193 | 0.727 |
| EM CD4+ T cells (%) | 37.30 (30.58-43.68) | 35.70 (28.40-45.70) | 37.00 (25.45-46.28) | 0.642 | 0.711 | 0.992 |
| EMRA CD4+ T cells (%) | 2.35 (1.20-3.90) | 1.30 (0.90-2.60) | 1.40 (1.10-2.20) | 0.040 | 0.007 | 0.881 |
| Treg cells (%) | 5.65 (4.95-7.20) | 5.70 (4.90-7.10) | 6.65 (5.10-8.70) | 0.855 | 0.144 | 0.334 |
| CD45RA+ Treg cells (%) | 21.25 (14.53-30.80) | 12.70 (7.30-17.50) | 13.15 (8.73-20.25) | <0.001 | <0.001 | 0.604 |
| Th17 cells (%) | 17.05 (14.35-19.50) | 19.90 (14.50-23.90) | 23.75 (17.85-30.25) | 0.243 | <0.001 | 0.011 |
| Th2 cells (%) | 31.30 (26.03-36.20) | 38.80 (32.00-48.50) | 37.35 (30.43-45.35) | 0.006 | 0.006 | 0.588 |
| Th1 cells (%) | 30.85 (26.18-36.00) | 25.40 (22.10-33.40) | 22.90 (14.90-28.00) | 0.084 | <0.001 | 0.042 |
| Tfh cells (%) | 14.65 (12.38-19.43) | 9.10 (7.90-17.20) | 12.80 (10.03-17.88) | 0.003 | 0.020 | 0.038 |
| Tfh17 cells (%) | 33.95 (30.93-39.00) | 27.70 (25.70-38.30) | 38.80 (31.45-46.10) | 0.022 | 0.015 | <0.001 |
| Tfh2 cells (%) | 30.05 (26.80-37.43) | 36.90 (31.40-47.50) | 33.05 (29.35-42.10) | 0.008 | 0.085 | 0.115 |
| Tfh1 cells (%) | 23.80 (19.60-27.53) | 22.40 (17.50-29.80) | 18.50 (14.10-23.45) | 0.590 | <0.001 | 0.005 |
| PD1+ Tfh cells (%) | 8.85 (5.83-12.58) | 17.70 (10.30-28.20) | 16.30 (10.80-25.55) | <0.001 | <0.001 | 0.753 |
| switched memory B cells (%) | 18.45 (13.65-22.70) | 20.40 (13.80-24.20) | 14.30 (7.50-24.35) | 0.496 | 0.088 | 0.060 |
| unswitched memory B cells (%) | 9.55 (7.33-15.08) | 5.30 (3.40-7.40) | 5.40 (3.00-7.95) | <0.001 | <0.001 | 0.978 |
| double negative B cells (%) | 4.20 (2.78-5.80) | 5.70 (3.60-9.30) | 6.60 (4.35-10.05) | 0.022 | <0.001 | 0.402 |
| naive B cells (%) | 67.75 (55.63-73.53) | 65.70 (56.90-73.50) | 69.40 (52.90-82.30) | 0.731 | 0.424 | 0.337 |
| plasma cells (%) | 0.65 (0.40-1.00) | 2.70 (1.50-6.70) | 2.35 (1.00-6.35) | <0.001 | <0.001 | 0.360 |
| NKG2D+ NK cells (%) | 50.20 (42.53-58.68) | 54.40 (29.60-68.00) | 40.55 (27.88-53.83) | 0.701 | 0.001 | 0.057 |
| NKG2A+ NK cells (%) | 29.45 (18.78-36.73) | 29.90 (16.90-44.50) | 26.90 (17.68-45.15) | 0.751 | 0.986 | 0.884 |
| perforin+ NK cells (%) | 92.95 (88.98-95.33) | 90.90 (84.90-94.80) | 90.05 (83.60-94.15) | 0.069 | 0.013 | 0.903 |
| granzyme B+ NK cells (%) | 93.15 (87.25-94.63) | 94.30 (90.50-97.60) | 93.90 (88.20-97.58) | 0.069 | 0.076 | 0.747 |
| HLA-DR+ monocytes (%) | 99.80 (99.40-99.90) | 95.20 (91.00-98.90) | 85.30 (55.68-96.23) | <0.001 | <0.001 | <0.001 |
| classic monocytes (%) | 93.35 (89.38-94.83) | 94.20 (91.30-97.40) | 93.70 (86.13-96.78) | 0.055 | 0.255 | 0.332 |
| intermediate monocytes (%) | 2.00 (1.38-3.03) | 2.30 (1.20-4.90) | 2.80 (1.40-9.63) | 0.414 | 0.025 | 0.285 |
| non-classic monocytes (%) | 5.15 (3.80-7.78) | 2.40 (0.80-4.60) | 2.00 (0.55-4.38) | <0.001 | <0.001 | 0.873 |
| HLA-DR MFI of monocytes | 8218.50 (6358.50-10302.25) | 2890.00 (2397.00-6638.00) | 1788.00 (715.00-4103.00) | 0.001 | <0.001 | 0.003 |
| DCs (%) | 0.80 (0.70-0.93) | 0.40 (0.30-0.60) | 0.20 (0.10-0.50) | <0.001 | <0.001 | 0.018 |
| CD86+ DCs (%) | 82.00 (75.40-87.80) | 85.30 (79.30-90.20) | 84.80 (74.30-91.00) | 0.147 | 0.151 | 0.884 |
| mDCs (%) | 85.95 (81.68-88.55) | 68.50 (55.70-82.80) | 41.45 (26.23-69.15) | <0.001 | <0.001 | 0.007 |
| pDCs (%) | 9.70 (7.18-20.53) | 3.50 (2.30-6.30) | 2.95 (1.20-5.95) | <0.001 | <0.001 | 0.569 |
| CD1c+ mDCs (%) | 33.40 (9.25-47.60) | 17.00 (11.50-45.60) | 17.40 (8.23-35.85) | 0.637 | 0.089 | 0.227 |
| CD141+ mDCs (%) | 1.95 (1.00-3.58) | 0.80 (0.50-2.00) | 2.95 (1.20-5.78) | 0.023 | 0.222 | 0.003 |
| M-MDSCs (%) | 0.00 (0.00-0.00) | 0.06 (0.02-0.38) | 0.82 (0.12-3.34) | <0.001 | <0.001 | <0.001 |
| PMN-MDSCs (%) | 0.01 (0.01-0.02) | 0.44 (0.14-0.83) | 0.45 (0.12-1.99) | <0.001 | <0.001 | 0.234 |
| PD-L1 (pg/mL) | 0.01 (0.01-1.70) | 10.22 (3.61-28.68) | 20.38 (3.61-71.49) | <0.001 | <0.001 | 0.357 |
| CCL2 (pg/mL) | 166.97 (90.59-217.70) | 179.34 (125.48-340.28) | 244.33 (127.60-403.73) | 0.097 | 0.001 | 0.346 |
| CCL3 (pg/mL) | 17.81 (2.13-28.42) | 36.37 (24.10-73.37) | 45.36 (22.75-92.27) | <0.001 | <0.001 | 0.371 |
| CCL4 (pg/mL) | 0.01 (0.01-0.01) | 0.01 (0.01-39.74) | 0.01 (0.01-118.20) | 0.427 | 0.008 | 0.113 |
| CD40L (pg/mL) | 2071.11 (1368.36-2878.39) | 1083.20 (418.87-2790.47) | 936.64 (133.67-1547.93) | 0.096 | <0.001 | 0.158 |
| CXCL10 (pg/mL) | 48.21 (26.82-68.39) | 85.56 (42.02-250.09) | 112.39 (65.58-236.67) | 0.004 | <0.001 | 0.389 |
| IL-8 (pg/mL) | 1.18 (0.13-5.71) | 15.58 (1.66-39.92) | 15.92 (3.36-62.00) | 0.002 | <0.001 | 0.380 |
| GM-CSF (pg/mL) | 8.42 (5.20-12.65) | 20.11 (9.46-30.69) | 22.17 (13.09-30.21) | <0.001 | <0.001 | 0.332 |
| granzyme B (pg/mL) | 0.01 (0.01-0.69) | 0.42 (0.01-3.14) | 0.01 (0.01-3.73) | 0.025 | 0.031 | 0.563 |
| IFN-a (pg/mL) | 0.92 (0.01-1.58) | 0.93 (0.28-1.38) | 0.82 (0.07-1.61) | 0.770 | 0.679 | 0.868 |
| IFN-g (pg/mL) | 0.01 (0.01-0.01) | 0.01 (0.01-0.01) | 0.01 (0.01-0.41) | 0.006 | 0.001 | 0.947 |
| IL-10 (pg/mL) | 46.66 (34.54-63.78) | 87.98 (46.58-118.17) | 122.53 (79.06-178.18) | 0.003 | <0.001 | 0.006 |
| IL-12p70 (pg/mL) | 0.01 (0.01-0.01) | 0.01 (0.01-0.01) | 0.01 (0.01-0.01) | 0.206 | 0.505 | 0.323 |
| IL-13 (pg/mL) | 4.47 (0.01-11.62) | 7.91 (0.01-12.32) | 5.34 (0.01-9.88) | 0.280 | 0.973 | 0.208 |
| IL-15 (pg/mL) | 1.35 (0.35-1.70) | 2.17 (1.01-3.24) | 2.55 (1.12-4.43) | 0.011 | <0.001 | 0.418 |
| IL-17A (pg/mL) | 0.01 (0.01-0.01) | 0.27 (0.01-1.53) | 0.01 (0.01-1.28) | <0.001 | <0.001 | 0.377 |
| IL-1b (pg/mL) | 0.01 (0.01-0.01) | 0.47 (0.01-1.15) | 0.01 (0.01-0.47) | <0.001 | <0.001 | 0.169 |
| IL-1ra (pg/mL) | 158.63 (119.94-266.36) | 971.49 (449.79-1844.79) | 1428.77 (749.25-2583.29) | <0.001 | <0.001 | 0.056 |
| IL-2 (pg/mL) | 0.01 (0.01-0.01) | 0.75 (0.30-1.46) | 0.75 (0.23-1.46) | <0.001 | <0.001 | 0.873 |
| IL-33 (pg/mL) | 0.01 (0.01-0.01) | 0.15 (0.01-1.81) | 0.15 (0.01-1.74) | 0.002 | <0.001 | 0.678 |
| IL-4 (pg/mL) | 0.01 (0.01-0.01) | 0.01 (0.01-0.01) | 0.01 (0.01-0.01) | 0.206 | 0.998 | 0.058 |
| IL-6 (pg/mL) | 0.50 (0.01-2.48) | 10.02 (5.40-17.01) | 15.66 (2.35-52.02) | <0.001 | <0.001 | 0.350 |
| TNF-a (pg/mL) | 0.01 (0.01-0.01) | 0.01 (0.01-1.77) | 0.01 (0.01-4.44) | 0.035 | <0.001 | 0.288 |
| IL-1a (pg/mL) | 0.01 (0.01-3.04) | 0.01 (0.01-0.01) | 0.01 (0.01-0.01) | 0.021 | 0.001 | 0.837 |
| Data are presented as median (25th - 75th percentile). HCs, healthy controls; WBC, white blood cells; NEU, neutrophils; LYM, lymphocytes; MON, monocytes; EOS, eosinophils; BASC, basophils; RBC, red blood cells; Hb, hemoglobin; PLT, platelet; ALT, alanine aminotransferase; AST, aspartate aminotransferase; TP, total protein; ALB, albumin; GLB, globulin; A/G, albumin/globulin; TBil, total bilirubin; DBil, direct bilirubin; IBil, indirect bilirubin; LDH, lactate dehydrogenase; Cre, Creatinine; PT, prothrombin time; PA, prothrombin activity; INR, international normalized ratio; FIB, fibrinogen; APTT, activated partial thromboplastin time; TT, thrombin time; NK, natural killer, CM, central memory; EM, effector memory; EMRA, terminally differentiated effector memory; Treg, regulatory T; Th, helper T; Tfh, follicular helper T; MFI, mean fluorescence intensity; DCs, dendritic cells; mDCs, myeloid DCs; pDCs, plasmacytoid DCs; M-MDSCs, monocytic-myeloid-derived suppressor cells; PMN-MDSCs, polymorphonuclear-myeloid-derived suppressor cells; CCL, C-C motif chemokine ligand; CD40L, CD40 Ligand; IL, interleukin; GM-CSF, granulocyte-macrophage colony stimulating factor; IFN, interferon; TNF, tumor necrosis factor. | | | | | | |

| **Supplementray Table 4. The results of immune indicators in sepsis patients with different outcomes.** | | | |
| --- | --- | --- | --- |
|  | Survived (n = 66) | Deceased (n = 24) | *p* |
| T cell count (/μl) | 608.87 (345.83-877.34) | 467.50 (254.87-720.50) | 0.183 |
| T cells (%) | 68.37 (59.06-77.85) | 63.28 (53.10-77.05) | 0.468 |
| CD8^+^ T cell count (/μl) | 211.50 (101.63-345.75) | 154.24 (89.37-342.37) | 0.559 |
| CD8^+^ T cells (%) | 21.67 (15.76-27.76) | 23.91 (13.47-36.65) | 0.523 |
| CD4^+^ T cell count (/μl) | 320.40 (195.58-557.75) | 248.50 (147.84-355.75) | 0.037 |
| CD4^+^ T cells (%) | 39.39 (29.48-51.39) | 34.53 (26.83-41.89) | 0.069 |
| NK cell count (/μl) | 89.00 (48.32-125.79) | 78.86 (39.32-105.02) | 0.399 |
| NK cells (%) | 8.66 (5.85-12.24) | 11.37 (6.65-22.29) | 0.19 |
| B cell count (/μl) | 150.13 (72.60-233.25) | 107.22 (52.90-172.55) | 0.369 |
| B cells (%) | 17.38 (9.96-24.43) | 15.61 (9.89-25.93) | 0.989 |
| CD4^+^ T cells/CD8^+^ T cells | 1.84 (1.15-2.67) | 1.58 (0.77-2.77) | 0.309 |
| HLA-DR^+^CD4^+^ T cells (%) | 18.05 (14.10-22.75) | 16.80 (8.95-21.95) | 0.276 |
| CD28^+^CD4^+^ T cells (%) | 96.05 (92.18-98.50) | 95.75 (92.10-99.13) | 0.909 |
| HLA-DR^+^CD8^+^ T cells (%) | 54.50 (42.38-68.70) | 50.20 (32.35-77.23) | 0.625 |
| CD28^+^CD8^+^ T cells (%) | 49.05 (34.13-67.50) | 49.70 (30.13-67.60) | 0.788 |
| HLA-DR^+^ T cells (%) | 34.35 (24.38-44.53) | 32.60 (23.78-51.38) | 0.993 |
| CM CD8^+^ T cells (%) | 2.95 (1.63-5.08) | 2.70 (1.48-4.28) | 0.532 |
| naive CD8^+^ T cells (%) | 12.90 (7.18-27.58) | 13.85 (6.40-21.05) | 0.82 |
| EM CD8^+^ T cells (%) | 43.20 (33.08-54.78) | 43.95 (35.60-48.40) | 0.685 |
| EMRA CD8^+^ T cells (%) | 31.80 (19.18-43.45) | 37.75 (23.53-46.25) | 0.457 |
| CM CD4^+^ T cells (%) | 25.85 (22.93-30.93) | 27.20 (20.43-33.05) | 0.888 |
| naive CD4^+^ T cells (%) | 31.85 (24.38-45.85) | 35.40 (19.33-48.48) | 0.749 |
| EM CD4^+^ T cells (%) | 37.70 (29.93-46.28) | 33.05 (21.75-45.28) | 0.32 |
| EMRA CD4^+^ T cells (%) | 1.40 (1.10-2.38) | 1.30 (0.98-1.85) | 0.261 |
| Treg cells (%) | 6.45 (4.95-8.00) | 7.10 (5.45-9.88) | 0.126 |
| CD45RA^+^ Treg cells (%) | 12.30 (8.53-17.88) | 14.05 (10.33-28.08) | 0.184 |
| Th17 cells (%) | 23.40 (17.85-28.05) | 28.10 (20.43-37.38) | 0.093 |
| Th2 cells (%) | 38.25 (29.70-44.90) | 36.80 (33.95-46.10) | 0.695 |
| Th1 cells (%) | 23.50 (17.80-27.98) | 15.70 (9.78-28.15) | 0.058 |
| Tfh cells (%) | 11.95 (9.83-17.88) | 13.90 (10.48-17.88) | 0.514 |
| Tfh17 cells (%) | 38.85 (33.30-43.60) | 38.45 (30.05-50.38) | 0.449 |
| Tfh2 cells (%) | 32.75 (29.35-41.78) | 33.45 (29.95-43.80) | 0.581 |
| Tfh1 cells (%) | 19.55 (14.80-23.25) | 16.95 (9.98-24.70) | 0.205 |
| PD1^+^ Tfh cells (%) | 15.60 (10.58-25.55) | 18.10 (12.75-24.13) | 0.446 |
| switched memory B cells (%) | 14.30 (5.88-23.93) | 13.95 (8.18-25.73) | 0.508 |
| unswitched memory B cells (%) | 4.60 (2.85-7.50) | 6.80 (4.45-13.93) | 0.033 |
| double negative B cells (%) | 6.15 (4.13-8.60) | 8.95 (4.93-14.80) | 0.065 |
| naive B cells (%) | 70.60 (59.63-83.33) | 62.90 (39.40-78.48) | 0.101 |
| plasma cells (%) | 2.20 (1.00-6.23) | 3.25 (0.83-6.33) | 0.902 |
| NKG2D^+^ NK cells (%) | 41.60 (30.43-54.35) | 36.15 (17.50-50.85) | 0.198 |
| NKG2A^+^ NK cells (%) | 30.95 (18.18-49.48) | 21.15 (13.15-29.43) | 0.012 |
| perforin^+^ NK cells (%) | 90.30 (84.93-94.05) | 88.90 (82.03-94.25) | 0.788 |
| granzyme B^+^ NK cells (%) | 92.90 (87.13-97.08) | 94.65 (91.68-98.10) | 0.122 |
| HLA-DR^+^ monocytes (%) | 90.20 (70.53-96.93) | 51.80 (34.98-70.25) | <0.001 |
| classic monocytes (%) | 93.85 (86.58-97.30) | 93.35 (83.38-96.38) | 0.452 |
| intermediate monocytes (%) | 2.45 (1.25-8.65) | 5.15 (2.35-11.20) | 0.038 |
| non-classic monocytes (%) | 2.05 (1.03-4.68) | 1.70 (0.20-2.98) | 0.178 |
| HLA-DR MFI of monocytes | 2581.00 (1241.50-5358.50) | 736.50 (481.00-1379.25) | <0.001 |
| DCs (%) | 0.25 (0.10-0.58) | 0.15 (0.00-0.43) | 0.07 |
| CD86^+^ DCs (%) | 85.25 (75.60-90.15) | 83.30 (72.35-92.28) | 0.953 |
| mDCs (%) | 44.30 (31.53-73.83) | 34.30 (10.18-50.45) | 0.016 |
| pDCs (%) | 3.05 (1.20-6.53) | 2.55 (1.15-4.90) | 0.391 |
| CD1c^+^ mDCs (%) | 17.00 (8.45-31.80) | 18.00 (7.03-38.70) | 0.767 |
| CD141^+^ mDCs (%) | 2.50 (1.05-4.60) | 3.80 (1.78-9.28) | 0.171 |
| M-MDSCs (%) | 0.38 (0.07-1.82) | 3.77 (0.86-6.00) | <0.001 |
| PMN-MDSCs (%) | 0.31 (0.11-1.32) | 1.65 (0.46-6.17) | 0.001 |
| PD-L1 (pg/mL) | 12.64 (0.20-42.70) | 52.81 (19.72-95.46) | 0.004 |
| CCL2 (pg/mL) | 199.74 (110.15-323.77) | 397.32 (200.38-944.92) | 0.005 |
| CCL3 (pg/mL) | 37.70 (20.59-88.01) | 65.42 (38.29-128.46) | 0.061 |
| CCL4 (pg/mL) | 0.01 (0.01-101.71) | 59.39 (0.01-155.14) | 0.020 |
| CD40L (pg/mL) | 936.64 (177.23-1547.93) | 1045.90 (0.01-1496.10) | 0.772 |
| CXCL10 (pg/mL) | 123.15 (67.36-242.49) | 101.21 (57.02-227.61) | 0.541 |
| IL-8 (pg/mL) | 11.82 (2.34-36.20) | 60.77 (32.75-150.88) | <0.001 |
| GM-CSF (pg/mL) | 21.24 (13.36-29.45) | 24.06 (10.18-37.23) | 0.685 |
| granzyme B (pg/mL) | 0.01 (0.01-3.52) | 0.01 (0.01-3.68) | 0.743 |
| IFN-α (pg/mL) | 0.71 (0.03-1.38) | 1.15 (0.49-3.37) | 0.027 |
| IFN-γ (pg/mL) | 0.01 (0.01-0.01) | 0.01 (0.01-0.61) | 0.236 |
| IL-10 (pg/mL) | 116.92 (79.06-165.06) | 133.06 (75.83-273.28) | 0.425 |
| IL-12p70 (pg/mL) | 0.01 (0.01-0.01) | 0.01 (0.01-0.01) | 0.546 |
| IL-13 (pg/mL) | 5.34 (0.01-9.88) | 7.91 (4.88-10.30) | 0.186 |
| IL-15 (pg/mL) | 2.05 (0.73-3.65) | 4.20 (2.58-5.64) | 0.002 |
| IL-17A (pg/mL) | 0.01 (0.01-1.28) | 0.10 (0.01-0.90) | 0.428 |
| IL-1β (pg/mL) | 0.01 (0.01-0.19) | 0.40 (0.16-1.08) | 0.001 |
| IL-1ra (pg/mL) | 1085.60 (653.37-2109.64) | 2383.51 (1559.28-3838.54) | 0.002 |
| IL-2 (pg/mL) | 0.52 (0.01-1.22) | 1.40 (1.16-2.59) | <0.001 |
| IL-33 (pg/mL) | 0.01 (0.01-0.89) | 1.38 (0.12-2.18) | 0.004 |
| IL-4 (pg/mL) | 0.01 (0.01-0.01) | 0.01 (0.01-0.01) | 0.998 |
| IL-6 (pg/mL) | 7.00 (1.11-36.58) | 43.87 (19.00-304.57) | <0.001 |
| TNF-α (pg/mL) | 0.01 (0.01-3.85) | 2.31 (0.01-8.59) | 0.033 |
| IL-1α (pg/mL) | 0.01 (0.01-0.01) | 0.01 (0.01-0.01) | 0.186 |
| Data are presented as number (%), or median (25th - 75th percentile). SOFA, sequential organ failure assessment; NK, natural killer, CM, central memory; EM, effector memory; EMRA, terminally differentiated effector memory; Treg, regulatory T; Th, helper T; Tfh, follicular helper T; MFI, mean fluorescence intensity; DCs, dendritic cells; mDCs, myeloid DCs; pDCs, plasmacytoid DCs; M-MDSCs, monocytic-myeloid-derived suppressor cells; PMN-MDSCs, polymorphonuclear-myeloid-derived suppressor cells; CCL, C-C motif chemokine ligand; CD40L, CD40 Ligand; IL, interleukin; GM-CSF, granulocyte-macrophage colony stimulating factor; IFN, interferon; TNF, tumor necrosis factor. | | | |

| **Supplementary Table 5. The accuracy of the predictive model based on 30 immune indicators measured in 90 patients with sepsis** | | | | |
| --- | --- | --- | --- | --- |
| **Predicted cluster** | **Original cluster** | | | **Class error** |
|  | **1** | **2** | **3** |  |
| **1** | 29 | 3 | 1 | 0.12 |
| **2** | 2 | 27 | 2 | 0.13 |
| **3** | 5 | 2 | 19 | 0.27 |
| The out-of-bag error estimate for the model was 16.67%. | | | | |

| **Supplementary Table 6. The accuracy of predictive model (based on 30 immune indicators) based on data from another cohort of 37 patients with sepsis.** | | | |
| --- | --- | --- | --- |
| **Predicted cluster** | **Original cluster** | | |
|  | **1** | **2** | **3** |
| **1** | 12 | 1 | 1 |
| **2** | 1 | 11 | 1 |
| **3** | 2 | 1 | 7 |
| The accuracy of the prediction model was 81.1% (95% CI: (64.8%, 92.0%)). | | | |


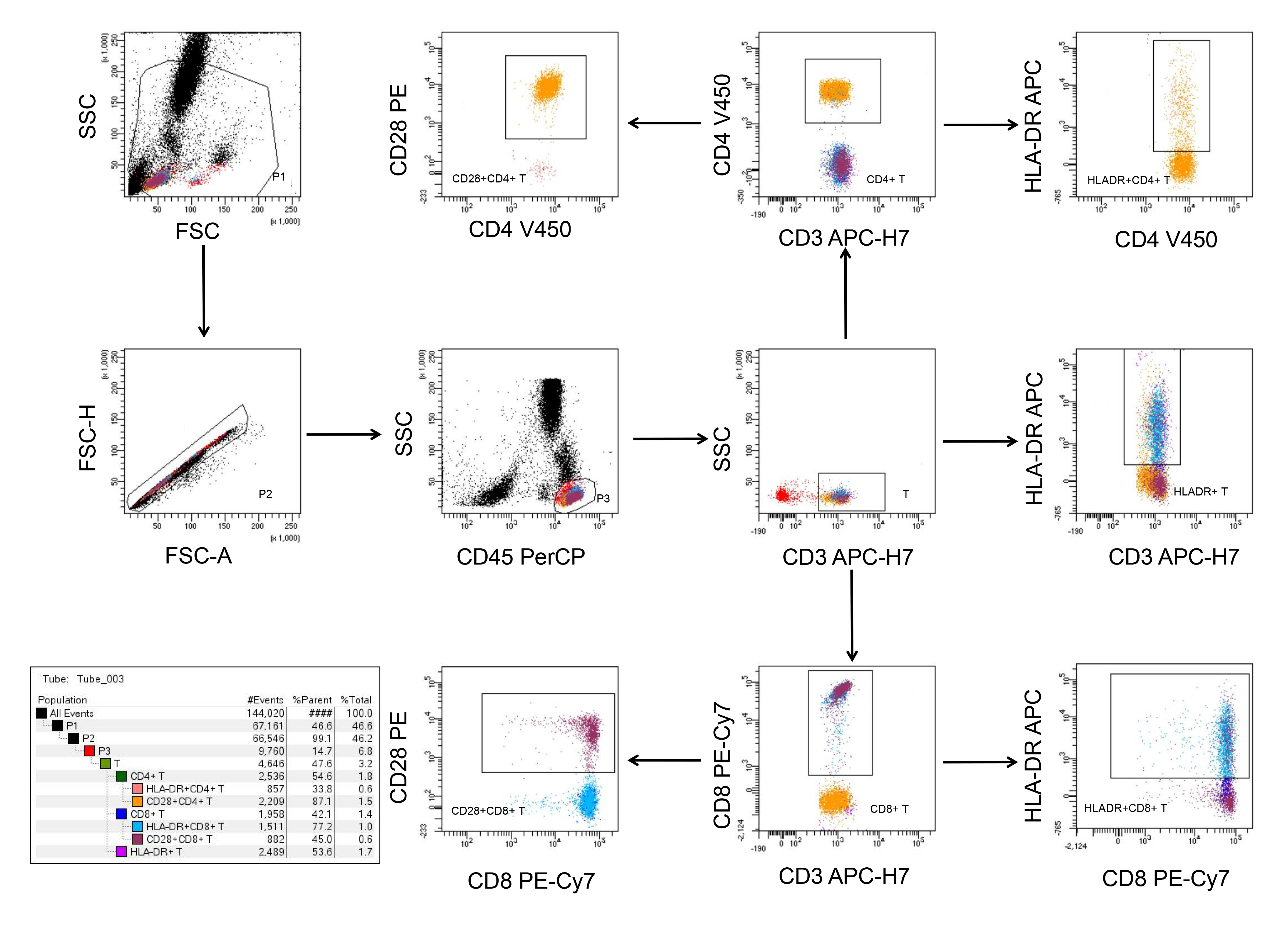


**Supplementary Figure 1**. Gating strategies of HLA-DR and CD28 expressions on CD4^+^ and CD8^+^ T cells.


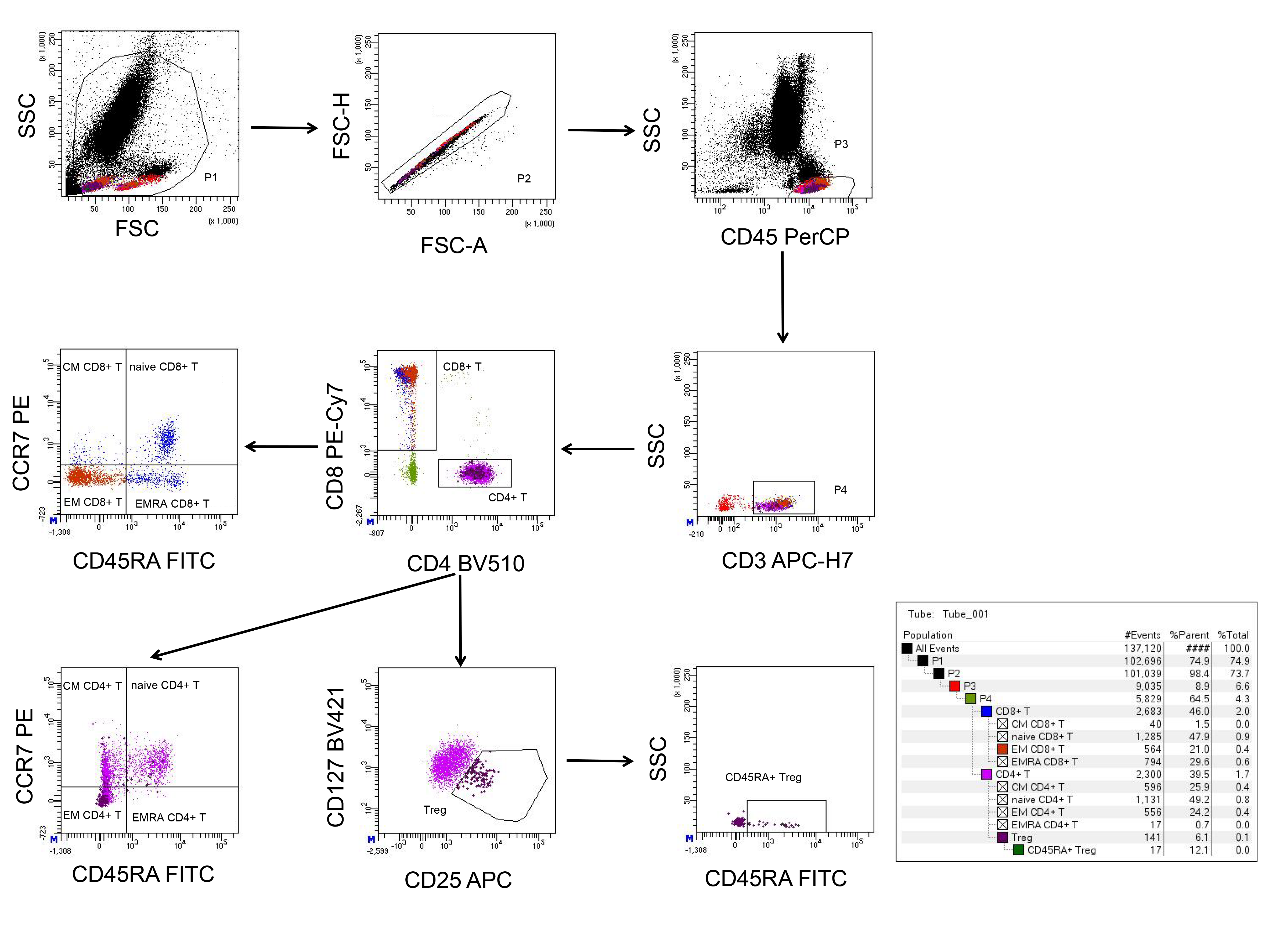


**Supplementary Figure 2**. Gating strategies of effector/memory subsets of T cells (CD45RA^+^CCR7^+^ naive cells, CD45RA^-^CCR7^+^ central memory (CM) cells, CD45RA^-^ CCR7^-^ effector memory (EM) cells, and CD45RA^+^CCR7^-^ terminally differentiated effector memory (EMRA) cells), and Treg cells (CD45RA^+^ naive Treg cells).


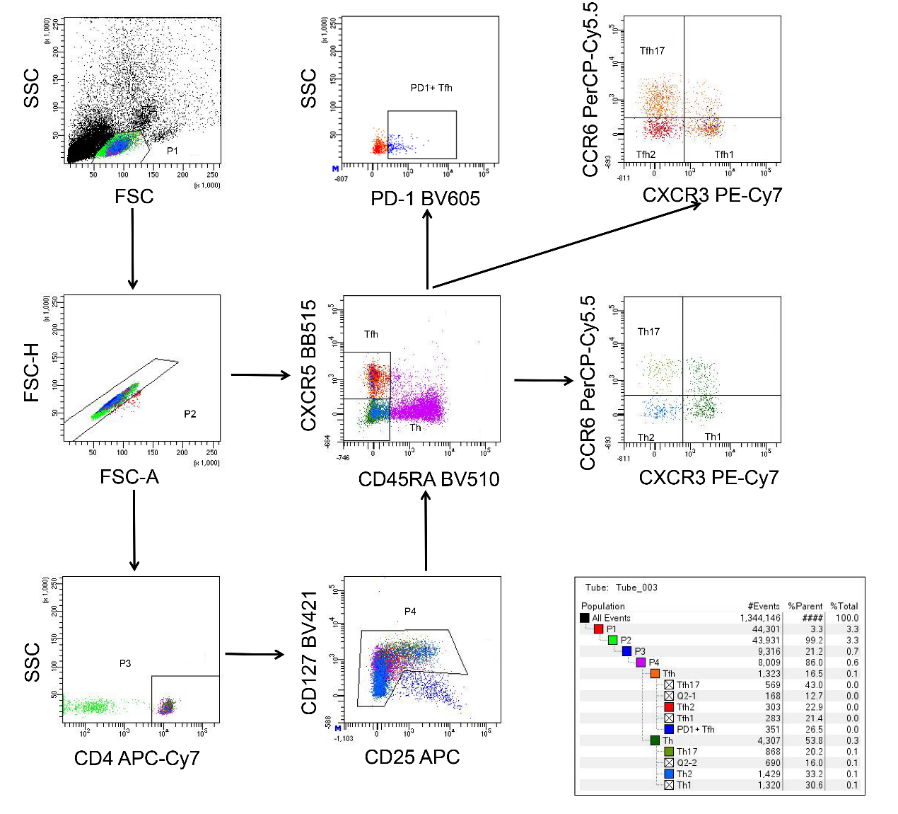


**Supplementary Figure 3**. Gating strategies of Th and Tfh cell subsets (CD4^+^CD25^low^CD127^high^CD45RA^-^CXCR5^-^ Th cells, CD4^+^CD25^low^CD127^high^CD45RA^-^CXCR5^-^CXCR3^+^CCR6^-^ Th1 cells, CD4^+^CD25^low^CD127^high^CD45RA^-^CXCR5^-^CXCR3^-^CCR6^-^ Th2 cells, CD4^+^CD25^low^CD127^high^CD45RA^-^CXCR5^-^CXCR3^-^CCR6^+^ Th17 cells, CD4^+^CD25^low^CD127^high^CD45RA^-^CXCR5^+^ Tfh cells, CD4^+^CD25^low^CD127^high^CD45RA^-^CXCR5^+^CXCR3^+^CCR6^-^ Tfh1 cells, CD4^+^CD25^low^CD127^high^CD45RA^-^CXCR5^+^CXCR3^-^CCR6^-^ Tfh2 cells, and CD4^+^CD25^low^CD127^high^CD45RA^-^CXCR5^+^CXCR3^-^CCR6^+^ Tfh17 cells).


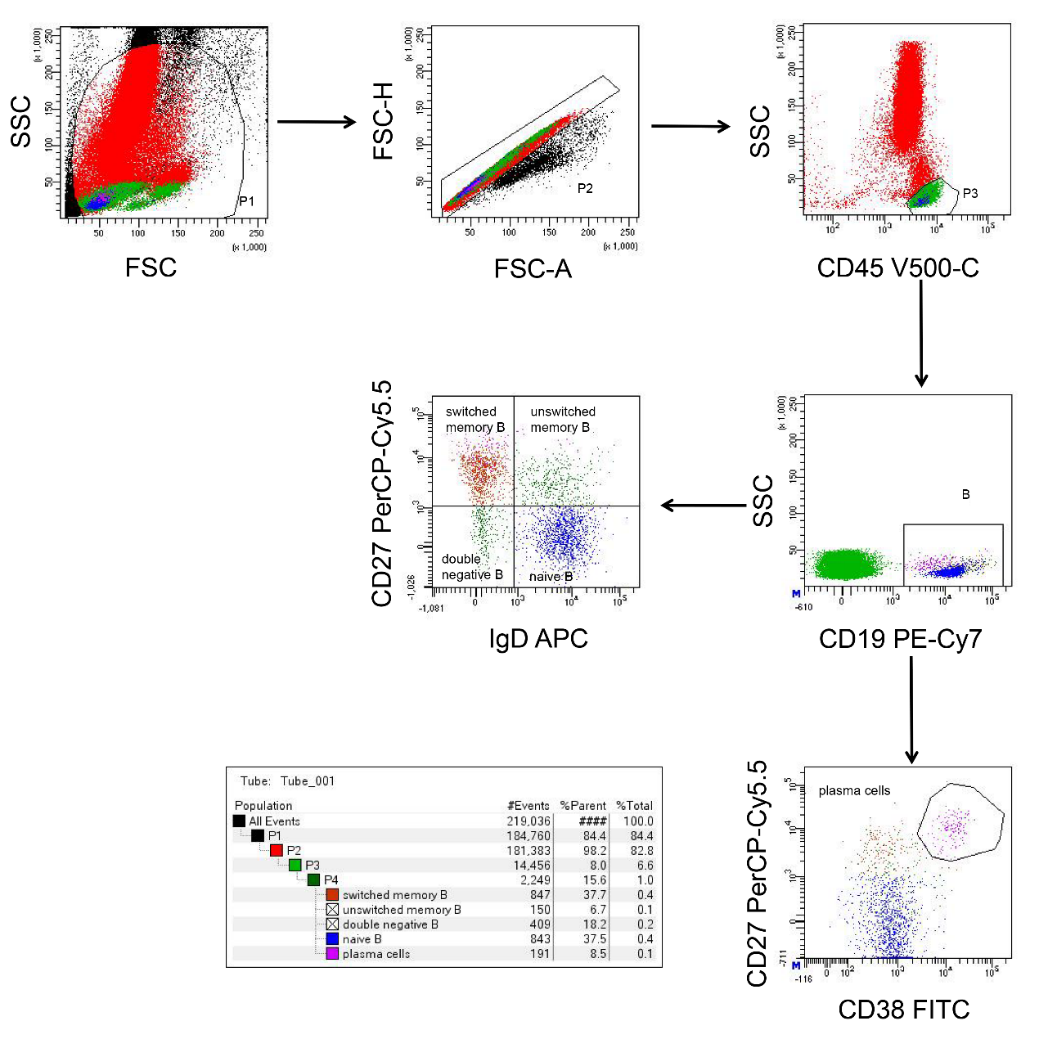


**Supplementary Figure 4**. Gating strategies of B cell subsets (CD19^+^IgD^+^CD27^-^ naive B cells, CD19^+^IgD^+^CD27^+^ unswitched memory B cells, CD19^+^IgD^-^CD27^+^ switched memory B cells, CD19^+^IgD^-^CD27^-^ double-negative B cells, and CD19^+^CD27^+^CD38^high^ plasma cells).


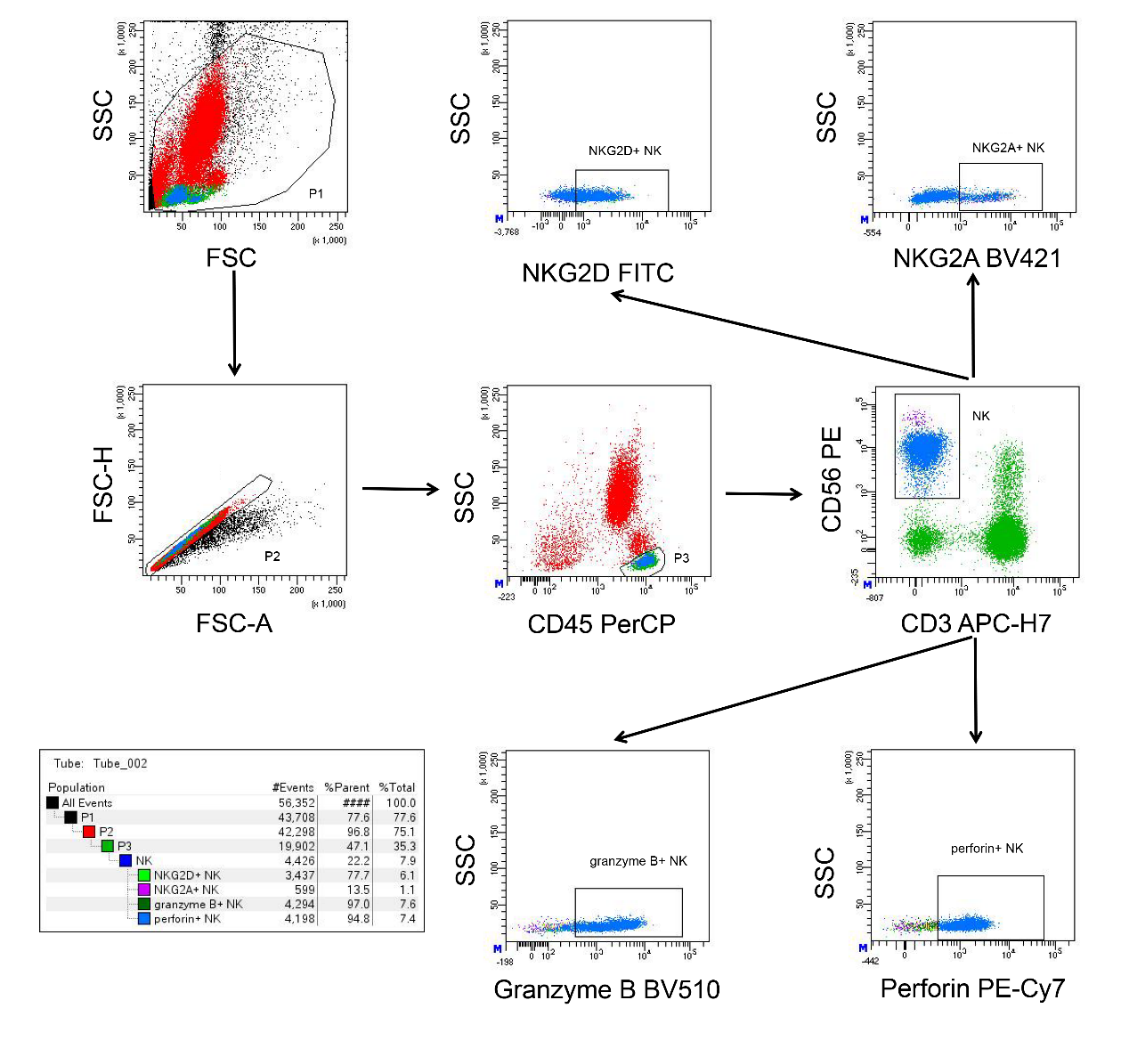


**Supplementary Figure 5**. Gating strategies of NK cell subsets (NKG2A^+^ NK cells, NKG2D^+^ NK cells, perforin^+^ NK cells, and granzyme B^+^ NK cells).


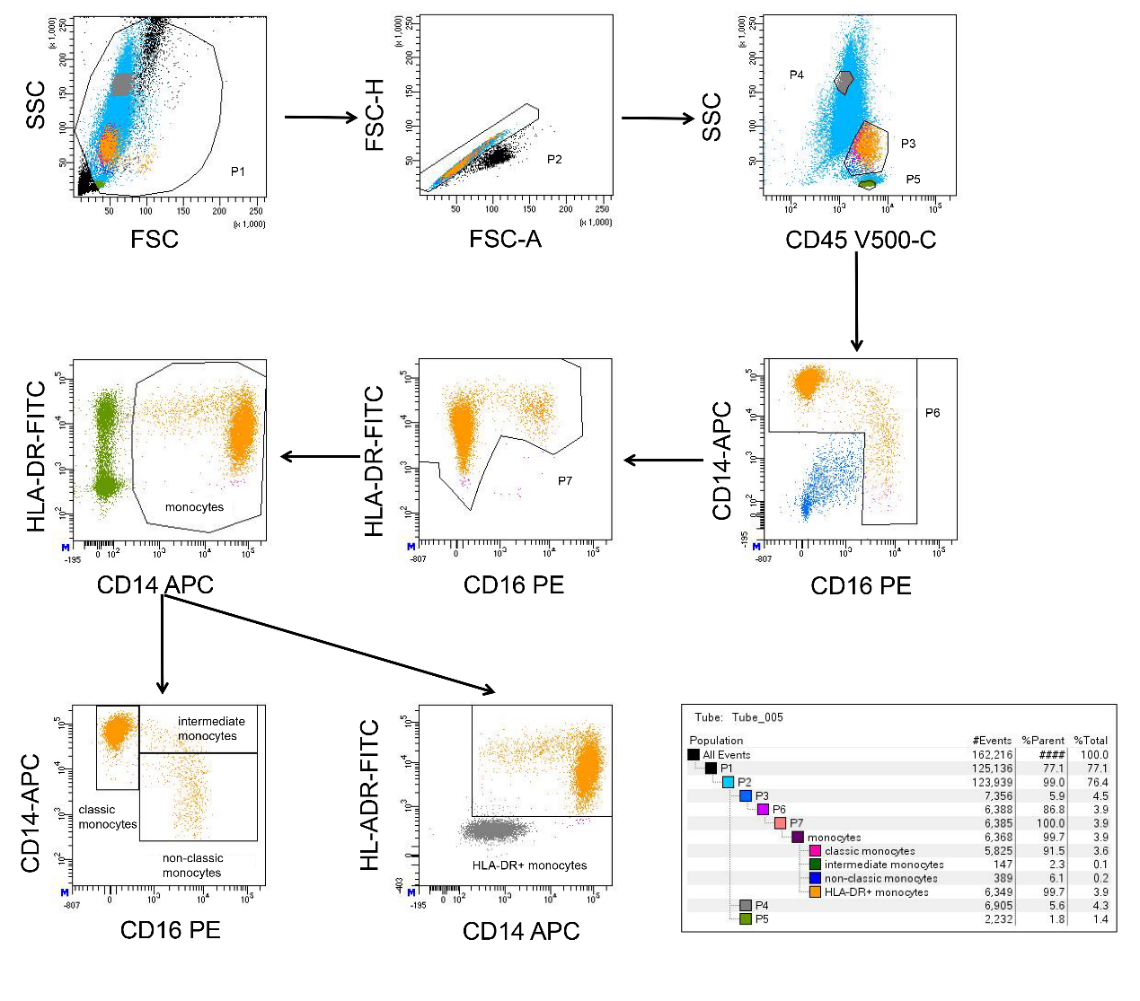


**Supplementary Figure 6**. Gating strategies of monocyte subsets (HLA-DR^+^CD14^++/+^ monocytes, CD16^-^CD14^++^ classic monocytes, CD16^+^CD14^++^ intermediate monocytes, and CD16^+^CD14^+^ non-classic monocytes).


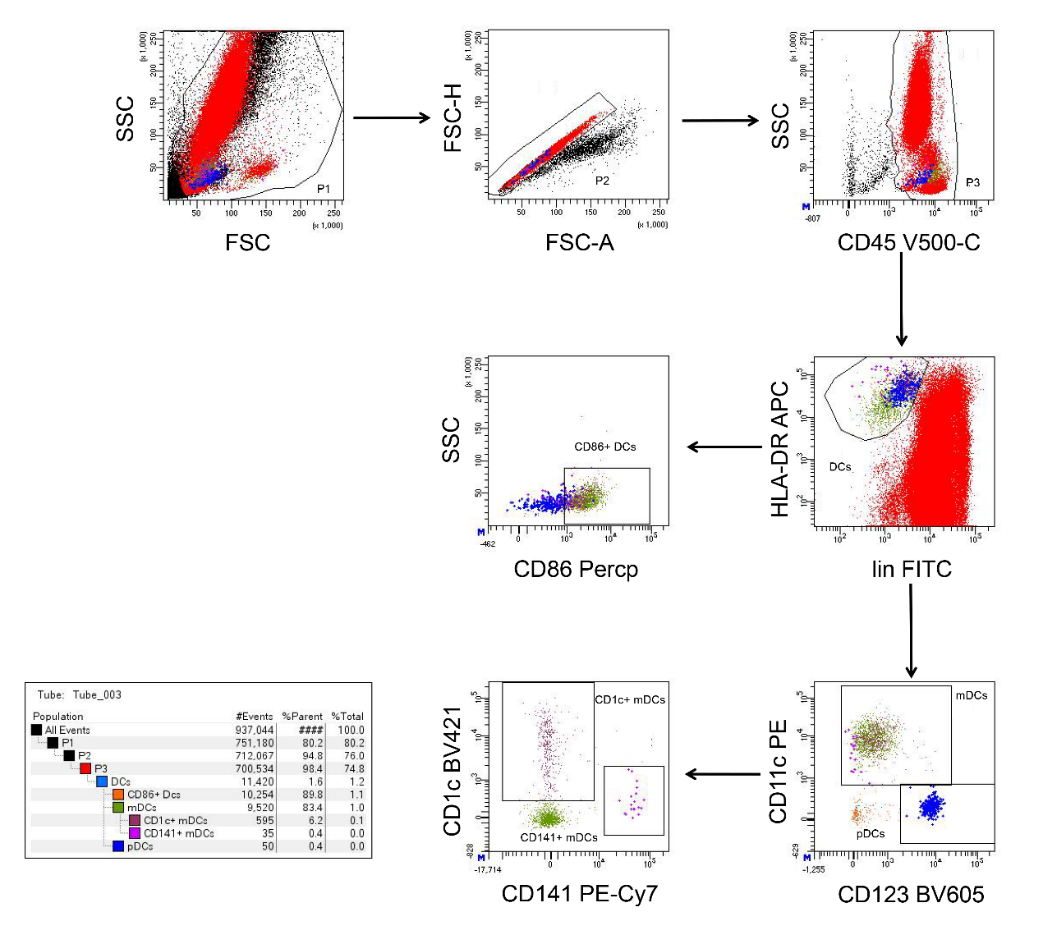


**Supplementary Figure 7**. Gating strategies of DC subsets (CD45^+^Lin^-^HLA-DR^+^ DCs, CD45^+^Lin^-^HLA-DR^+^CD86^+^ DCs, CD45^+^Lin^-^HLA-DR^+^CD123^+^ plasmacytoid DCs (pDCs), CD45^+^Lin^-^HLA-DR^+^CD11c^+^ myeloid DCs (mDCs), CD45^+^Lin^-^HLA-DR^+^CD11c^+^CD1c^+^ mDCs, and CD45^+^Lin^-^HLA-DR^+^CD11c^+^CD141^+^ mDCs).


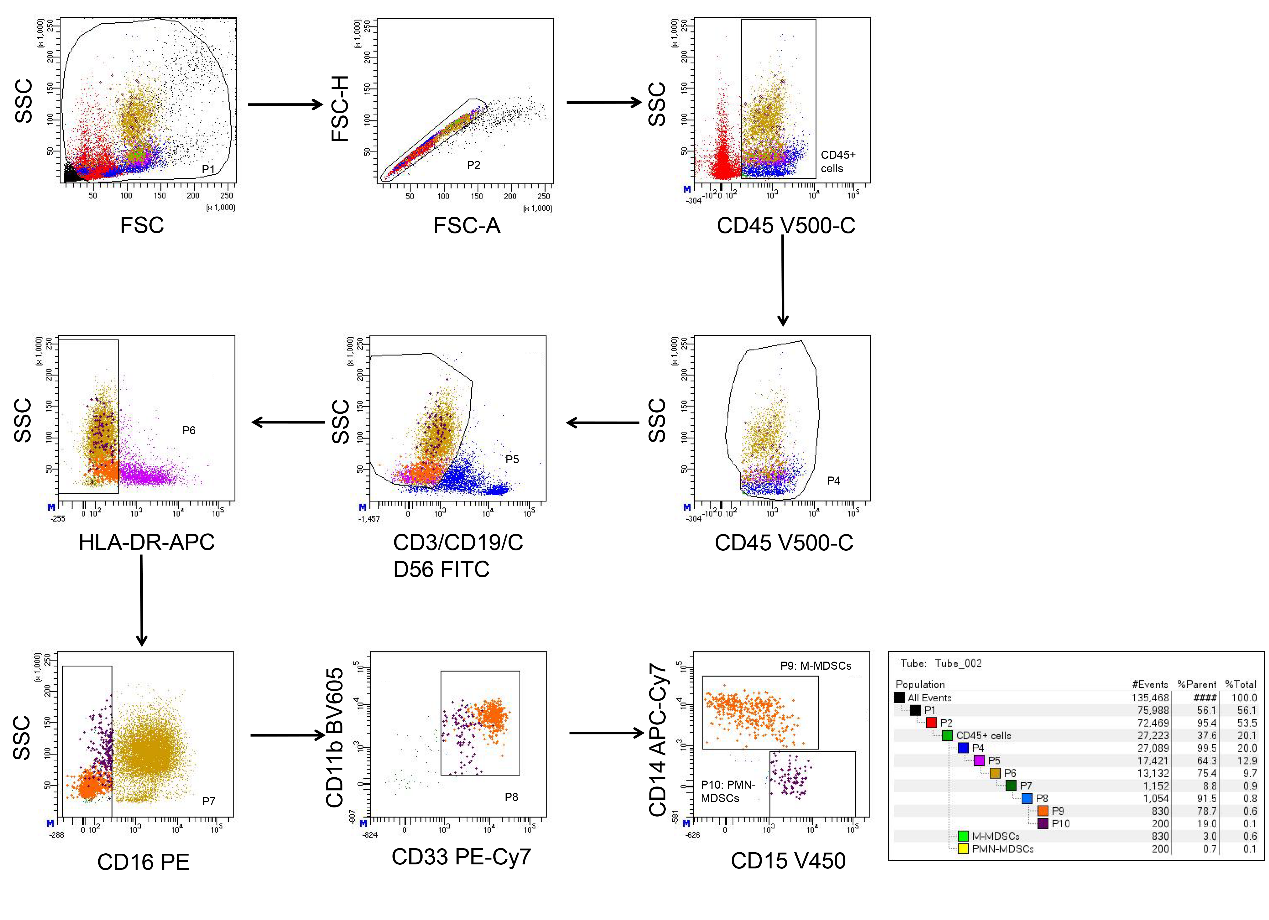


**Supplementary Figure 8**. Gating strategies of MDSC subsets (CD14^+^CD15^-^CD16^-^HLA-DR^-/low^CD11b^+^CD33^+^ monocytic-MDSCs (M-MDSCs), and CD15^+^CD14^-^CD16^-^HLA-DR^-/low^CD11b^+^CD33^+^ polymorphonuclear-MDSCs (PMN-MDSCs)).


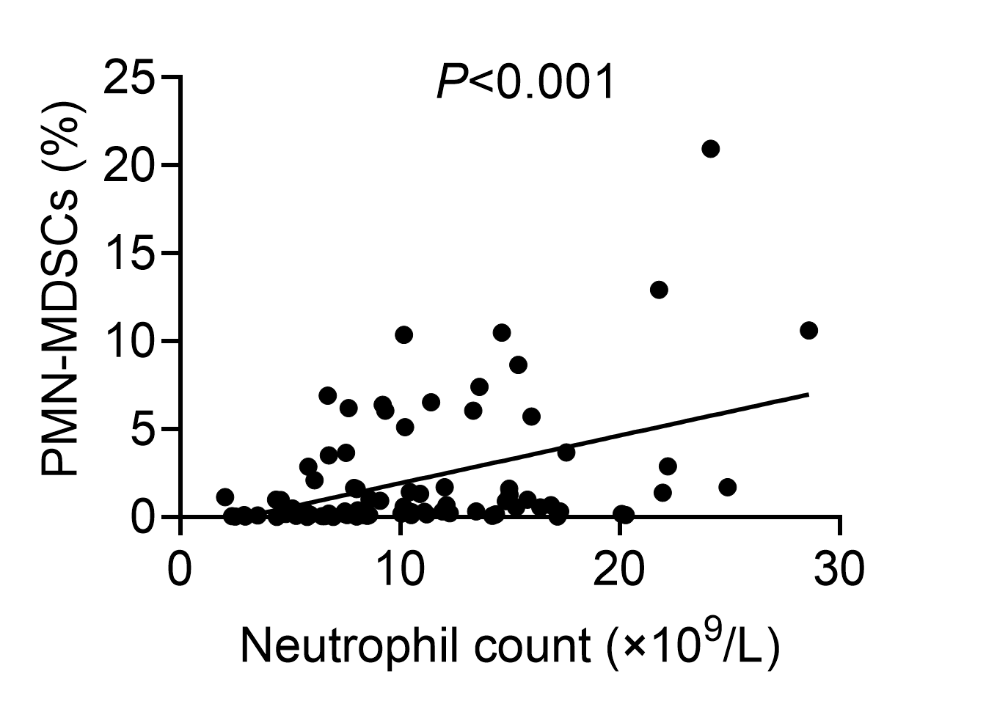


**Supplementary Figure 9**. The correlation analysis between PMN-MDSCs and neutrophils."
